# Stimuli‐Responsive and Defect‐Regulated Luminescent Organic Metal Halide for High‐Security Anti‐Counterfeiting and Force Sensing

**DOI:** 10.1002/advs.202510163

**Published:** 2025-07-12

**Authors:** Chunyan Jiang, Jing Yan, Rongkai Du, Yang Li, Mingmei Wu, Beibei Xu, Jianrong Qiu

**Affiliations:** ^1^ State Key Laboratory of Extreme Photonics and Instrumentation College of Optical Science and Engineering Zhejiang University Hangzhou 310027 China; ^2^ Zhuhai Key Laboratory of Optoelectronic Functional Materials and Membrane Technology School of Chemical Engineering and Technology Sun Yat‐sen University Zhuhai 519082 China; ^3^ Institute of Light+X Science and Technology Faculty of Electrical Engineering and Computer Science Ningbo University Ningbo 315211 China

**Keywords:** defect, organic metal halide, stimuli‐responsive luminescence, tunable luminescence

## Abstract

Smart materials with stimuli‐responsive luminescence hold considerable potential for anti‐counterfeiting and sensing applications. However, achieving multimode‐responsive luminescence and luminescence modulation remains a significant challenge. Herein, a smart 0D organic metal halide hybrid (OMH) (C_9_NH_20_)_6_[Pb_3_Br_12_] exhibiting temperature/moisture/mechanical force‐responsive luminescence switching and tunable responsive luminescence color is reported. The heat‐activated luminescence switching originates from a crystal structure transformation involving small molecule extraction. The recovery of the transformed structure under cooling and ambient conditions exposure through small molecule reinsertion is highly sensitive to moisture. Interestingly, defects in the recovered structure can be controlled, bringing a pathway to tune the luminescence color. Moreover, 0D (C_9_NH_20_)_6_[Pb_3_Br_12_] exhibits self‐recovering mechanical force‐responsive luminescence switching driven by structure deformation. Based on these stimuli‐responsive properties, its applications in time‐dependent high‐security anti‐counterfeiting and handwriting recognition are demonstrated. This study not only provides new insights for designing smart stimuli‐responsive luminescent OMH materials but also highlights the potential of (C_9_NH_20_)_6_[Pb_3_Br_12_] as a versatile platform for advanced anti‐counterfeiting and force sensing applications.

## Graphical abstract

A smart 0D (C_9_NH_20_)_6_[Pb_3_Br_12_], exhibiting temperature/moisture/mechanical force‐responsive luminescence and tunable luminescence color, is developed. Crystal structure transformation, defect, and structure deformation are systematically clarified as the origins of this responsive luminescence behavior. The material demonstrates promising applications in time‐dependent high‐security anti‐counterfeiting and handwriting recognition systems.

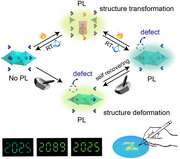

## 1 Introduction

Stimuli‐responsive luminescent materials, which exhibit luminescence switching in response to external stimuli, have garnered considerable attention as a prominent category of smart materials.^[^ ^1^, ^2^, ^3^, ^4^ ^]^ Based on the type of stimuli, they can be classified into various categories, including humidity‐, pH‐, heat‐, light‐, electricity‐, magnetism‐, and mechanical force‐responsive luminescent materials.^[^ ^5^, ^6^, ^7^, ^8^, ^9^, ^10^, ^11^ ^]^ As the terminology implies, stimuli‐responsive luminescent material offers promising applications in sensing.^[^ ^12^, ^13^, ^14^, ^15^ ^]^ In addition, their responsiveness makes them ideal candidates for advanced anti‐counterfeiting technologies. The widespread prevalence of counterfeit goods in the market poses a significant threat to property safety, social stability, and even human health, underscoring the urgency for the development of anti‐counterfeiting technologies.^[^ ^16^, ^17^ ^]^ Traditional luminescent security tags, which rely on static luminescence triggered by UV or near‐infrared light, are increasingly inadequate in meeting the evolving demands of modern security protocols.^[^ ^18^, ^19^, ^20^ ^]^ In contrast, smart stimuli‐responsive luminescent materials provide security tags with dynamic and adaptable properties, significantly enhancing the level of information security and fitting perfectly into the contemporary landscape where counterfeiters continuously challenge existing security safeguards.^[^ ^21^, ^22^, ^23^ ^]^

With outstanding optoelectronic properties, including high defect tolerance, strong absorption coefficient, long carrier diffusion length, and excellent luminescent properties, OMHs have emerged as one of the most prominent materials, offering promising applications in solar cells, light‐emitting diodes, scintillators, and photoelectric detectors.^[^ ^24^, ^25^, ^26^, ^27^ ^]^ However, their intrinsic ionic structure, characterized by low formation energy, renders them highly susceptible to structural transitions when exposed to external stimuli such as light, heat, solvents, and moisture.^[^ ^28^ ^]^ Although this property can be detrimental to devices, it paradoxically endows OMHs with the versatility for facile synthesis and the potential to evolve into stimuli‐responsive luminescent materials.^[^ ^29^, ^30^, ^31^ ^]^ Extensive research has been conducted to explore the stimuli‐responsive behaviors of OMHs, focusing on reversible and irreversible switching behaviors driven by changes in crystal structure, composition, and phase.^[^ ^32^ ^]^ Key mechanisms include: 1) interactions with H_2_O, solvents, and gas molecules that induce structural alterations;^[^ ^33^, ^34^ ^]^ 2) anion exchange in CsPbBr_3‐x_I_x_, resulting in mixed or separated phases;^[^ ^35^, ^36^ ^]^ 3) solid‐state reactions;^[^ ^37^, ^38^ ^]^ 4) phase transitions or glass transitions;^[^ ^39^, ^40^ ^]^ and 5) dynamic processes of crystal lattice construction and decomposition.^[^ ^41^ ^]^ Nevertheless, potential defect formation in the switching process has received little attention. On the other hand, among various stimuli, heat and moisture stand out as readily accessible stimuli sources for optical anti‐counterfeiting applications. However, most existing OMH materials are limited to two switching luminescence colors, which significantly constrains their confidentiality.^[^ ^42^, ^43^, ^44^ ^]^ Additionally, while there is growing interest in stimuli‐responsive OMHs, few studies have explored the luminescence switching responding to mechanical force stimuli,^[^ ^45^, ^46^ ^]^ despite its pivotal importance for applications in smart displays and sensing.

In this study, we developed a multimodal stimuli‐responsive and defect‐regulated luminescent OMH material for multifunctional applications. A non‐emissive 0D bulk (C_9_NH_20_)_6_[Pb_3_Br_12_] single crystal was synthesized via the assembly of C_9_NH_20_Br with PbBr_2_. Upon heating, the material undergoes luminescence switching accompanied by a crystal structure transformation. Subsequent cooling and exposure to moisture induce the reversion of structure at a relative humidity‐dependent rate. However, the recovered structure exhibits distinct luminescent properties compared to the pristine single crystal. The luminescence color of the recovered structure can be tuned by adjusting the C_9_NH_20_Br content. Furthermore, (C_9_NH_20_)_6_[Pb_3_Br_12_] demonstrates self‐recovering luminescence switching under mechanical force stimulation. Systematic investigations of structure changes and luminescent properties under varying temperature and pressure conditions suggest that structure deformation serves as the fundamental mechanism. This work reveals defect formation during the switching process and its ability to regulate luminescence, thereby overcoming the conventional two‐color limitation in stimuli‐responsive systems. Leveraging these stimuli‐responsive luminescence properties, we demonstrated advanced time‐dependent high‐security anti‐counterfeiting and handwriting recognition applications using (C_9_NH_20_)_6_[Pb_3_Br_12_].

## 2 Results and Discussion

### 2.1 Heat/Moisture‐Responsive Luminescence Switching

**Figure 1:**
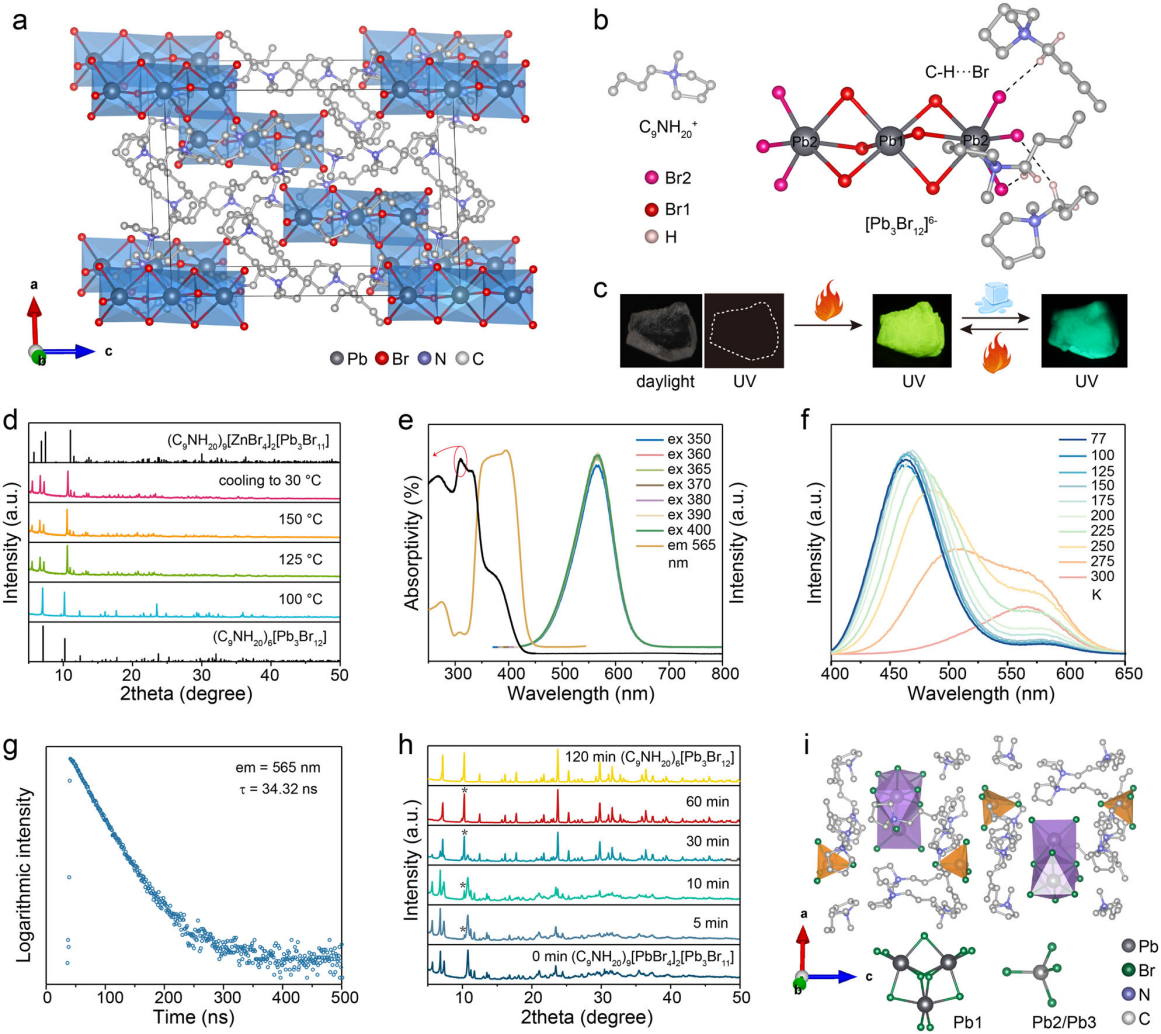
a) Crystal structure of (C_9_NH_20_)_6_[Pb_3_Br_12_] (hydrogen atoms omitted for clarity). b) View of [Pb_3_Br_12_]^6−^ cluster and its coordination with C_9_NH_20_^+^ via C‐H···Br. c) Photographs of (C_9_NH_20_)_6_[Pb_3_Br_12_] single crystal under daylight and UV light, after heating‐cooling under UV light, and after heating‐cooling followed by ambient conditions exposure under UV light. d) Variable‐temperature PXRD patterns of (C_9_NH_20_)_6_[Pb_3_Br_12_] during a heating and cooling cycle. e) PL excitation/emission, absorption spectra, f) temperature‐dependent PL emission spectra, and g) time‐resolved PL spectrum of the heat‐converted (C_9_NH_20_)_9_[PbBr_4_]_2_[Pb_3_Br_11_]. h) Time‐dependent PXRD patterns of the heat‐converted (C_9_NH_20_)_9_[PbBr_4_]_2_[Pb_3_Br_11_] under ambient conditions exposure. i) Crystal structure of (C_9_NH_20_)_9_[PbBr_4_]_2_[Pb_3_Br_11_] (hydrogen atoms omitted for clarity).

A transparent, colorless, plate‐like single crystal of (C_9_NH_20_)_6_[Pb_3_Br_12_] was synthesized by the temperature‐lowering method. Its crystal structure was determined from single‐crystal X‐ray diffraction data. (C_9_NH_20_)_6_[Pb_3_Br_12_] crystallizes in the $R\bar{3}$ space group with a = b = 17.331(2) Å, c = 21.787(2) (1) Å, and more detailed crystal parameters are provided in Table S1 (Supporting Information). The crystal structure (**Figure** **1**a) features molecular‐level 0D architecture where linear [Pb_3_Br_12_]^6−^ trimers are completely isolated by C_9_NH_20_^+^ cations. Two crystallographically distinct Pb sites are observed (Figure 1b): Pb1 exhibits an almost regular octahedral coordination with a bond length of d(Pb ‐ Br) = 3.0176(1) Å, Pb2 displays a distorted octahedral coordination with a broader range of Pb‐Br bond lengths ranging from 2.9214(1) Å to 3.2430(1) Å. Each [Pb1Br6] octahedron is connected with two [Pb2Br6] octahedra via face‐sharing, forming an isolated linear [Pb2Br6–Pb1Br6–Pb2Br6] cluster. The structure contains a single crystallographically independent C_9_NH_20_^+^ cation. Each [Pb_3_Br_12_]^6−^ cluster interacts with six C_9_NH_20_^+^ cations through C─H···Br hydrogen bonds, with terminal [Pb2Br6] octahedron coordinating three C_9_NH_20_^+^ cations. Phase purity and crystallinity of the synthesized single crystals were confirmed by powder X‐ray diffraction (PXRD). Its diffraction peaks match well with those simulated from single‐crystal data, indicating the high purity and good crystallinity (Figure S1, Supporting Information).

The bulk (C_9_NH_20_)_6_[Pb_3_Br_12_] single crystal is non‐emissive under UV light at room temperature (RT). However, when heated to 130 °C, the bulk crystal pulverizes and emits yellow luminescence, which is well preserved after cooling back to RT. With increasing exposure time under ambient conditions, the yellow luminescence gradually switches to the green luminescence (Figure 1c). To elucidate the luminescence switching of (C_9_NH_20_)_6_[Pb_3_Br_12_] during heating, its structural evolution was investigated through variable‐temperature PXRD (Figure 1d). The room‐temperature diffraction patterns perfectly match the simulated results from the (C_9_NH_20_)_6_[Pb_3_Br_12_] single crystal. Upon heating, the diffraction peaks shift toward lower angles due to lattice expansion. At 125 °C, the initial diffraction peaks disappear while new peaks emerge, indicating a crystal structure transition. The transformed diffraction pattern shows high consistency with (C_9_NH_20_)_9_[ZnBr_4_]_2_[Pb_3_Br_11_], with only a minor angular shift. The transformed crystal structure remains stable upon cooling from 150 °C to RT, consistent with the observed luminescence changes. We further characterized the optical properties of the yellow‐emitting sample through absorption and photoluminescence (PL) spectra (Figure 1e). The broad emission exhibits a slightly asymmetrical peak shape with a central wavelength of 560 nm and a full width at half maximum (FWHM) of 85 nm. The excitation spectrum exhibits two minor bands at 279 and 310 nm, along with prominent excitation maxima at 350 and 380 nm. The absorption band in the range of 250–450 nm corresponds well to the excitation band, indicating the intrinsic emission nature.^[^ ^47^, ^48^ ^]^ Identical emission profiles under different excitation wavelengths suggest the presence of a single luminescent center. To further shed light on the photophysical properties of the yellow luminescence, temperature‐dependent PL emission spectra at low temperatures and time‐resolved PL spectrum were analyzed. A new high‐energy emission emerges at 464 nm at 77 K, whose intensity is stronger than that of the low‐energy emission at 576 nm (Figure 1f). As the temperature increases, the high‐energy emission undergoes a red shift, and the intensity of both emissions increases slightly. At 200 K, a significant decrease in the high‐energy emission and a considerable increase in the low‐energy emission are observed. When the temperature exceeds 275 K, the intensity of both high‐energy and low‐energy emissions decreases considerably. The emission profile predominantly consists of low‐energy emission centered at 565 nm at RT, and its time‐resolved PL spectrum reveals a single exponential decay with a lifetime of 34.32 ns (Figure 1g).

Since attempts to synthesize single crystal of (C_9_NH_20_)_9_[PbBr_4_]_2_[Pb_3_Br_11_] were unsuccessful, (C_9_NH_20_)_9_(ZnBr_4_)_2_[Pb_3_Br_11_] was synthesized according to a previous report for comparison.^[^ ^49^ ^]^ Its purity was confirmed by its PXRD pattern (Figure S2, Supporting Information). (C_9_NH_20_)_9_(ZnBr_4_)_2_[Pb_3_Br_11_] exhibits an emission spectrum similar to that of the heat‐converted crystal structure, with slight shifts in wavelength and changes in peak width (Figure S3, Supporting Information). It displays a broad emission with a peak at 567 nm and a FWHM of 69 nm. The PL lifetime of 567 nm emission is 33.43 ns, close to that of the heat‐converted crystal structure (Figure S4, Supporting Information). Temperature‐dependent emission spectra of the heat‐converted crystal structure are typical of [Pb_3_X_11_]^5−^ trimer cluster (Figure 1f), as observed in (C_9_NH_20_)_9_(MnBr_4_)_2_[Pb_3_Br_11_] and (C_9_NH_20_)_9_(ZnCl_4_)_2_[Pb_3_Cl_11_].^[^ ^50^, ^51^ ^]^ These results confirm that the (C_9_NH_20_)_6_[Pb_3_Br_12_] transforms into (C_9_NH_20_)_9_[PbBr_4_]_2_[Pb_3_Br_11_] upon heating, and elucidate the origin of its luminescence. The temperature‐dependent dual emission is attributed to two triplet energy minima on the excited‐state potential energy surface arising from the distortion of the trimer cluster (Figure S5, Supporting Information). When the temperature increases from low temperature to RT, the excitons generated after excitation can readily overcome the energy barrier between the high‐energy and low‐energy emitting states, resulting in a luminescence evolution from blue to yellow. DSC curve of (C_9_NH_20_)_6_[Pb_3_Br_12_] reveals an endothermic peak at 120 °C, corresponding to the structure transformation process (Figure S6, Supporting Information). Thermal gravimetric analysis shows that weight loss due to decomposition begins at 250 °C (Figure S7, Supporting Information), demonstrating the high stability of the structure.

The heat‐switched yellow luminescence gradually changes to green under ambient conditions over time. Time‐dependent PXRD patterns of this transformation process (Figure 1h) reveal that the diffraction peaks of (C_9_NH_20_)_6_[Pb_3_Br_12_] emerge after 5 min and intensify progressively with prolonged exposure. After 120 min, the pattern matches perfectly with pure‐phase (C_9_NH_20_)_6_[Pb_3_Br_12_] without impurity peaks, confirming the reversible structural transformation during the heating‐cooling‐exposure cycle. The chemical equilibrium can be expressed as Equation (1):

(1) $$\begin{array}{cc} & (5{\left(C_{9}NH_{20}\right)}_{6}\left[Pb_{3}Br_{12}\right])\underset{\mathrm{moisture}}{\overset{\mathrm{heat}}{⇌}}3{\left(C_{9}NH_{20}\right)}_{9}{\left[\mathrm{PbB}r_{4}\right]}_{2}\left[Pb_{3}Br_{11}\right]\\ & \;+3C_{9}NH_{20}\mathrm{Br} \end{array}$$

XRD pattern of the transformed (C_9_NH_20_)_9_[PbBr_4_]_2_[Pb_3_Br_11_] shows excellent agreement with the simulated pattern derived from the single‐crystal structure of (C_10_NH_22_)_9_[PbBr_4_]_2_[Pb_3_Br_11_] (Figure S8, Supporting Information),^[^ ^52^ ^]^ confirming their identical crystallization in the *P*31*c* space group. The absence of reference C_9_NH_20_Br diffraction peaks in the transformed structure may originate from their weak intensity, a phenomenon analogous to the reported [Bzmim]_3_SbCl_6_‐to‐[Bzmim]_2_SbCl_5_ transformation (Bzmim = *1*‐Benzyl‐*3*‐Methylimidazolium).^[^ ^53^ ^]^ The crystal structure of (C_9_NH_20_)_9_[PbBr_4_]_2_[Pb_3_Br_11_] (Figure 1i) also reveals completely isolated 0D structure where triangular [Pb_3_Br_11_]^5−^clusters and tetrahedral [PbBr_4_]^2−^ units are separated by C_9_NH_20_^+^ cations. Three distinct Pb coordination environments exist: 1) Pb1 coordinates with six Br atoms, with three such octahedra sharing faces to form fused triangular [Pb_3_Br_11_]^5−^clusters; 2) Pb2 and Pb3 each coordinate with four Br atoms, forming two discrete [PbBr_4_]^2−^ tetrahedra. Therefore, the structural transformation involves the following coordination changes: each linear [Pb_3_Br_12_]^6−^ cluster converts into 0.6 fused [Pb_3_Br_11_]^5−^ clusters and 1.2 isolated [PbBr_4_]^2−^ (Figure S9, Supporting Information).

### 2.2 Tunable Responsive Luminescence Color

**Figure 2:**
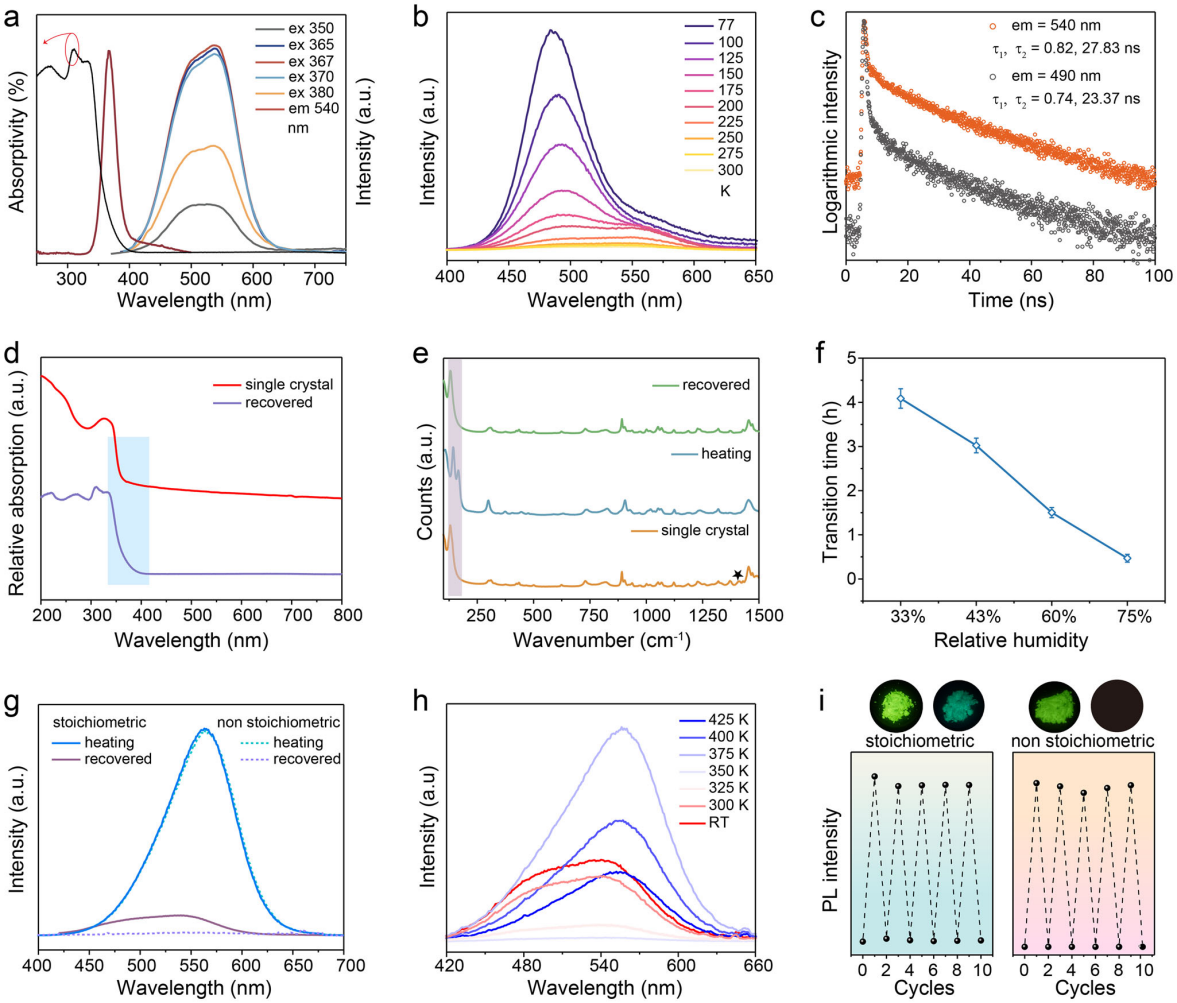
a) PL excitation/emission, absorption spectra, b) temperature‐dependent PL emission spectra, and c) time‐resolved PL spectra of the humidity‐recovered (C_9_NH_20_)_6_[Pb_3_Br_12_] powder. d) Absorption spectra of (C_9_NH_20_)_6_[Pb_3_Br_12_] single crystal and recovered (C_9_NH_20_)_6_[Pb_3_Br_12_] powder. e) Raman spectra of (C_9_NH_20_)_6_[Pb_3_Br_12_] single crystal, heat‐converted (C_9_NH_20_)_9_[PbBr_4_]_2_[Pb_3_Br_11_], and recovered (C_9_NH_20_)_6_[Pb_3_Br_12_] powder. f) Recovery time for the heat‐converted (C_9_NH_20_)_9_[PbBr_4_]_2_[Pb_3_Br_11_] to revert to (C_9_NH_20_)_6_[Pb_3_Br_12_] as a function of relative humidity. g) PL emission spectra of heat‐converted structure and recovered structure of stoichiometric and nonstoichiometric (C_9_NH_20_)_6_[Pb_3_Br_12_] (nonstoichiometric (C_9_NH_20_)_6_[Pb_3_Br_12_] contains surplus C_9_NH_20_Br). h) Temperature‐dependent PL emission spectra of green‐emitting (C_9_NH_20_)_6_[Pb_3_Br_12_] during the heating process. i) PL intensity variation of stoichiometric and nonstoichiometric (C_9_NH_20_)_6_[Pb_3_Br_12_] under 10 cycles of heating‐cooling‐exposure.

To investigate the origin of luminescence changes between single crystal and recovered powder (same crystal structures), we first examined the optical properties, including absorption and PL spectra, of the green‐emitting sample (**Figure** **2**a). The excitation spectrum displays a main band at 370 nm and a side band within the range of 400–500 nm. Unlike the heat‐converted (C_9_NH_20_)_9_[PbBr_4_]_2_[Pb_3_Br_11_], which exhibits almost consistent excitation and absorption bands, this green‐emitting sample exhibits a significant red shift (370 nm excitation vs 337 nm absorption) with an extended long wavelength excitation tail. Emission spectra under different excitation wavelengths reveal two overlapping centers with peaks at 540 and 490 nm, with the peak separation becoming more pronounced upon excitation wavelength tuning from 350 to 380 nm. To further understand the luminescence centers of the green‐emitting sample, temperature‐dependent PL emission spectra at low temperatures were analyzed (Figure 2b). The integral emission intensity shows a slight enhancement with the temperature decreasing from 300 to 200 K. When the temperature is further reduced from 200 to 77 K, the 540 nm emission continues to increase slightly, while the 490 nm band undergoes significant intensification and blue shifts to 483 nm. The short PL lifetimes (τ_1_ = 2.11 ns and τ_2_ = 6.51 ns, Figure S10, Supporting Information) of this 483 nm emission suggest that it may arise from organic components,^[^ ^54^ ^]^ as confirmed by the comparative PL emission spectrum and time‐resolved PL spectrum of C_9_NH_20_Br. Under excitation at 382 nm, the emission spectrum of C_9_NH_20_Br consists of an asymmetric broad band with a maximum at 451 nm (Figure S11, Supporting Information). Its lifetimes (τ_1_ = 1.47 ns and τ_2_ = 5.47 ns) are comparable to that of 483 nm emission from the green‐emitting sample (Figure S12, Supporting Information). The observed spectral shifts between isolated and framework‐incorporated C_9_NH_20_^+^ species reflect a modified chemical environment.^[^ ^55^ ^]^ Time‐resolved PL spectra of the green‐emitting sample at RT (Figure 2c) reveal bi‐exponential decay for both emissions (540 nm: τ_1_ = 0.82 ns, τ_2_ = 27.83 ns; 490 nm: τ_1_ = 0.74 ns and τ_2_ = 23.37 ns). The lifetime differences of the organic component between 77 K (483 nm) and RT (490 nm) arise from the merging of higher‐energy and low‐energy emissions at increasing temperatures. The non‐overlapping excitation and absorption spectra, along with the long‐wavelength excitation tail, indicate green luminescence from defects.

To verify this hypothesis, we compared the absorption spectra of a pristine (C_9_NH_20_)_6_[Pb_3_Br_12_] single crystal and recovered (C_9_NH_20_)_6_[Pb_3_Br_12_] powder (Figure 2d). The (C_9_NH_20_)_6_[Pb_3_Br_12_] powder exhibits a distinct red shift in the band edge relative to the single crystal, indicating bandgap narrowing due to defect‐induced subgap states. Raman spectroscopy of three samples: 1) pristine (C_9_NH_20_)_6_[Pb_3_Br_12_] single crystal, 2) heat‐converted (C_9_NH_20_)_9_[PbBr_4_]_2_[Pb_3_Br_11_], and 3) humidity‐recovered (C_9_NH_20_)_6_[Pb_3_Br_12_] powder (Figure 2e), confirm structure modification during heating conversion (100–250 cm^−1^ region changes). In contrast, the humidity‐recovered (C_9_NH_20_)_6_[Pb_3_Br_12_] powder maintains framework integrity, exhibiting identical vibration modes to the pristine (C_9_NH_20_)_6_[Pb_3_Br_12_] single crystal except for a 1413 cm⁻¹ peak attributable to glass substrate fluorescence under 785 nm laser excitation (due to the transparency of single crystal). Details of the structural transformation were further investigated. The recovery from converted (C_9_NH_20_)_9_[PbBr_4_]_2_[Pb_3_Br_11_] to (C_9_NH_20_)_6_[Pb_3_Br_12_] is highly sensitive to moisture (Figure 2f). Under a low relative humidity of 11%, the recovery does not occur for several days. Under ambient conditions with varying humidity (maintained by saturated salt solutions), the response time differs dramatically: at 75%, 60%, 43%, and 33% relative humidity, the recovery completion takes 0.47, 1.51, 3.01, and 4.08 h, respectively. Normalized emission spectra of recovered (C_9_NH_20_)_6_[Pb_3_Br_12_] (Figure S13, Supporting Information) demonstrate humidity‐dependent defect emission, with the emission intensity of the 540 nm band increasing at lower relative humidity. The recovery of high‐temperature converted structure at 100% relative humidity leads to the formation of another crystal structure, (C_9_NH_20_)_2_Pb_2_Br_6_ (Table S2, Supporting Information). This structure also forms when (C_9_NH_20_)_6_[Pb_3_Br_12_] is exposed to 100% relative humidity.

Additional evidence for the defect origin of green luminescence is provided by solvent‐evaporation‐synthesized (C_9_NH_20_)_9_[PbBr_4_]_2_[Pb_3_Br_11_]. By heating an acetonitrile solution containing C_9_NH_20_Br and PbBr_2_ in a 4:1 molar ratio (non‐stoichiometric for (C_9_NH_20_)_6_[Pb_3_Br_12_]), we successfully obtained (C_9_NH_20_)_9_[PbBr_4_]_2_[Pb_3_Br_11_], as confirmed by PXRD (Figure S14, Supporting Information). This synthesis was performed without protective ligands, and a 2:1 molar ratio of C_9_NH_20_Br to PbBr_2_ yielded impure products. The solvent‐evaporation‐synthesized (C_9_NH_20_)_9_[PbBr_4_]_2_[Pb_3_Br_11_] exhibits contrasting luminescence switching behavior. Its yellow luminescence reverts to a non‐emissive state within minutes of environmental exposure (Figure 2g), matching the non‐emissive nature of the pristine (C_9_NH_20_)_6_[Pb_3_Br_12_] single crystal. PXRD analysis confirmed that the recovered sample matches (C_9_NH_20_)_6_[Pb_3_Br_12_] (Figure S15, Supporting Information). Notably, when mixing C_9_NH_20_Br with PbBr_2_, their contact interface immediately exhibits yellow emission under UV light, with moisture exposure enhancing this luminescence. Combined with the humidity‐sensitive structure recovery (Figure 2f) and humidity‐dependent emission in recovered structure (Figure S13, Supporting Information), these phenomena demonstrate that the reactivity of C_9_NH_20_Br is strongly humidity‐dependent—solvated C_9_NH_20_Br can enhance Br^−^ migration by weakening electrostatic attraction, thereby increasing reactivity.^[^ ^56^ ^]^ Defect formation originates from incomplete C_9_NH_20_Br incorporation, as not all C_9_NH_20_Br molecules are reactive. With excessive C_9_NH_20_Br, its strong hygroscopicity both elevates system reactivity and ensures sufficient reactant quantities, facilitating defect‐free (C_9_NH_20_)_6_[Pb_3_Br_12_] formation with single‐crystal‐consistent luminescence. This defect model also explains the discrepancies with the prior work from Xia's group:^[^ ^57^ ^]^ their reported (C_9_NH_20_)_6_[Pb_3_Br_12_] single crystal exhibits green luminescence. Apart from a minor blue shift in the low‐energy emission region, their PL spectra closely resemble our results. Through femtosecond transient absorption (fs‐TA) spectroscopy and DFT calculations, they confirmed the green luminescence stems from defect‐trapped excitons. Under optical excitation, the trapped holes and electrons undergo radiative recombination. Therefore, for (C_9_NH_20_)_6_[Pb_3_Br_12_], defects may arise either during synthesis or post‐processing. In their study, defects formed during experimental procedures, while in our case, defects originated from incomplete lattice recovery. To further identify the specific nature of defects, the Br and Pb environments in both single crystal and recovered (C_9_NH_20_)_6_Pb_3_Br_12_ were investigated using XPS (Figure S16, Supporting Information). The Br 3d peaks can be fitted into two peaks with binding energies of 64.3 and 65.2 eV for single crystal and recovered (C_9_NH_20_)_6_Pb_3_Br_12_, corresponding to inner and surface Br^−^ ions, respectively.^[^ ^58^ ^]^ The intensity ratio between inner and surface ions decreases in the recovered (C_9_NH_20_)_6_Pb_3_Br_12_, which can be attributed to an increase in the Br^−^ content on the surface of recovered (C_9_NH_20_)_6_Pb_3_Br_12_. The lower binding energy shift observed in recovered (C_9_NH_20_)_6_Pb_3_Br_12_ results from an increase in electron cloud density around Pb due to the formation of Br vacancies. Notably, although the recovered structure exhibits green emission distinct from the non‐emissive nature of the pristine single crystal, it retains the heat‐responsive luminescence switching behavior. Temperature‐dependent PL emission spectra of the green‐emitting (C_9_NH_20_)_6_[Pb_3_Br_12_] sample present that the green emission intensity gradually decreases with increasing temperature and becomes nearly completely quenched at 350 K due to strong electron‐phonon coupling (Figure 2h). Upon further heating to 375 K, a broad emission band centered at 565 nm emerges (originating from (C_9_NH_20_)_9_[PbBr_4_]_2_[Pb_3_Br_11_]), achieving a switching to yellow emission. After cooling from 425 K to room temperature, the yellow emission persists with its intensity enhanced by 24‐fold compared to that at 425 K (Figure S17, Supporting Information), owing to reduced thermal quenching effects.

The luminescence intensity of C_9_NH_20_Br is extremely weak and virtually disappears upon hygroscopic absorption at RT, rendering its influence on the emission properties of both the heat‐converted (C_9_NH_20_)_9_[PbBr_4_]_2_[Pb_3_Br_11_] and recovered (C_9_NH_20_)_6_[Pb_3_Br_12_] negligible. The reversibility of structural transformation and luminescence switching in (C_9_NH_20_)_6_[Pb_3_Br_12_] was systematically evaluated through cycling experiments (Figure 2i). Both stoichiometric and non‐stoichiometric (C_9_NH_20_)_6_[Pb_3_Br_12_] samples exhibit subtle variations in emission intensity and wavelength after 10 heating‐cooling‐exposure cycles (Figures S18 and S19, Supporting Information), demonstrating not only excellent reversible luminescence switching but also stable defect formation during structural transformations under constant humidity conditions. Furthermore, neither measurable changes in PL lifetimes (Figures S20 and S21, Supporting Information) nor detectable alterations in the crystal structure (Figures S22 and S23, Supporting Information) were observed after multiple cycles, further confirming the exceptional reversibility of this system. The emission quantum yields of yellow‐emitting (C_9_NH_20_)_9_(PbBr_4_)_2_[Pb_3_Br_11_] and green‐emitting (C_9_NH_20_)_6_[Pb_3_Br_12_] are 31% and 7%, respectively, values that are not outstanding among OMH luminescent switching materials.^[^ ^31^, ^39^, ^40^ ^]^ However, these materials exhibit higher stability than CsPbX_3_ perovskites.^[^ ^59^ ^]^ Additionally, the fast heat‐responsive structure transformation, slow moisture‐involved recovery, and defect formation enable unique luminescence switching in (C_9_NH_20_)_6_[Pb_3_Br_12_].

### 2.3 Mechanical Force‐Responsive Luminescence Switching

**Figure 3:**
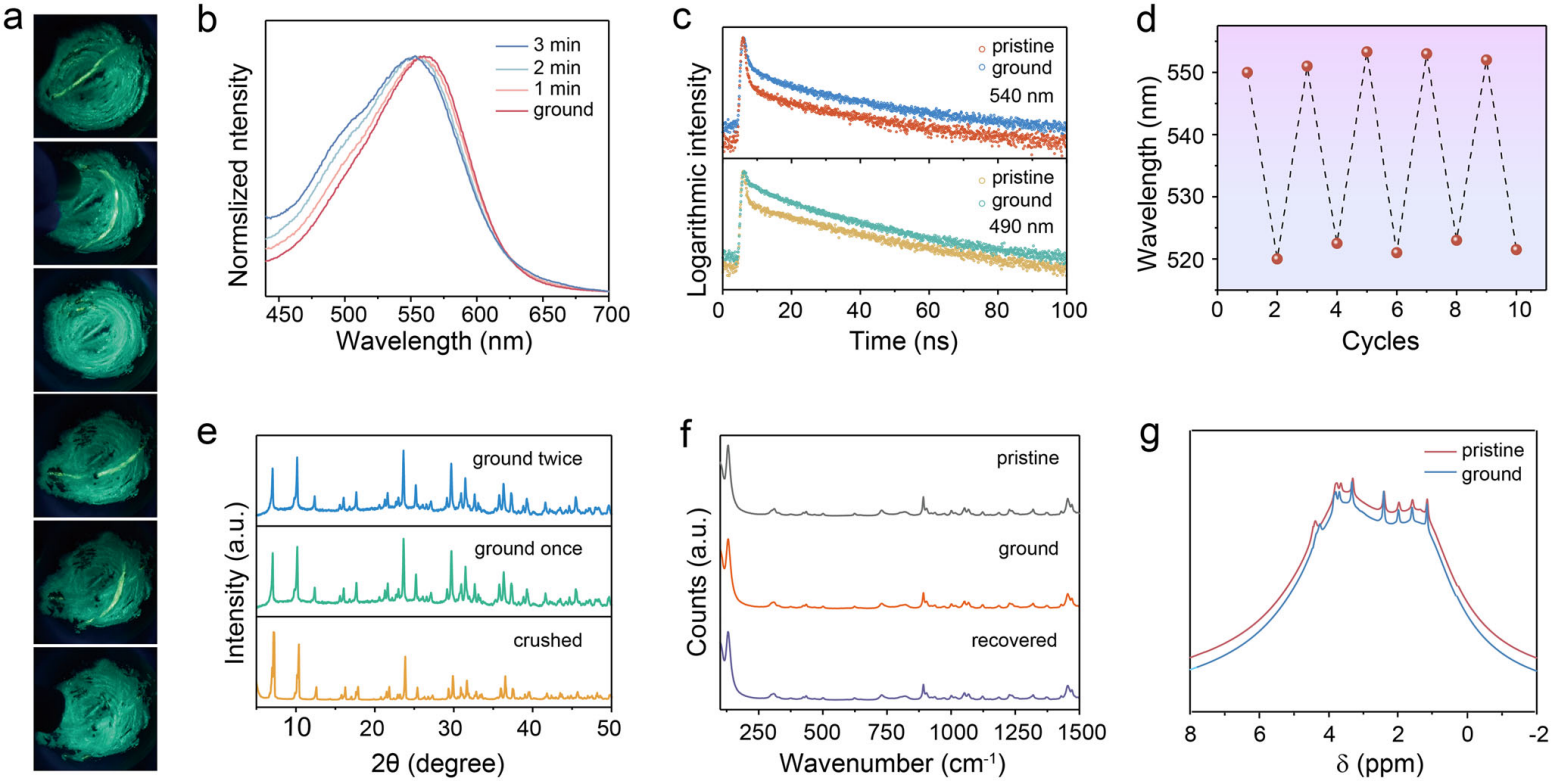
a) Photographs depicting the luminescence switching of (C_9_NH_20_)_6_[Pb_3_Br_12_] upon grinding under UV light. The luminescence turns into yellow after grinding and self‐recovers to green after ≈3 min. b) Normalized PL emission spectra of (C_9_NH_20_)_6_[Pb_3_Br_12_] during the grinding and self‐recovery processes. c) Comparison of PL decay curves of the 540 and 490 nm emission of the pristine and ground (C_9_NH_20_)_6_[Pb_3_Br_12_]. d) Emission wavelength variation under 10 cycles of grinding and recovery. e) XRD patterns of the crushed (C_9_NH_20_)_6_[Pb_3_Br_12_] and the (C_9_NH_20_)_6_[Pb_3_Br_12_] with two rounds of grinding. f) Raman spectra of the pristine, ground, and post‐grinding recovered (C_9_NH_20_)_6_[Pb_3_Br_12_]. g) Magnified NMR H^1^ spectra of the pristine and ground (C_9_NH_20_)_6_[Pb_3_Br_12_].

The bulk (C_9_NH_20_)_6_[Pb_3_Br_12_] single crystal is non‐emissive. However, when crushed into small crystals using a mortar and pestle, yellow luminescence inevitably appears in certain regions and swiftly transforms into green luminescence. Upon further grinding, all contact areas exhibit yellow luminescence, which fades into green within a few minutes after grinding cessation. Ultimately, the entire powder sample exhibits uniform green luminescence. Remarkably, the mechanical force‐responsive luminescence switching behavior is maintained in the green‐emitting powder sample (denoted as pristine sample in subsequent descriptions), which presents yellow luminescence during grinding and spontaneously recovers to its green luminescence within minutes (**Figure** **3**a; Video S1, Supporting Information). The evolution of normalized and unnormalized emission spectra during grinding (Figure 3b; Figure S24, Supporting Information) demonstrates that grinding enhances the emission intensity of both low‐energy and high‐energy bands, with faster growth and a red shift observed for the low‐energy emission. As time after cessation of grinding increases, the emission intensity of both bands gradually decreases, and the low‐energy emission decreases at a faster rate than the high‐energy emission. Within 3 min, the emission spectrum recovers to closely resemble that of the green‐emitting sample obtained after the heating‐cooling‐exposure, except for a slightly increased low‐energy emission. PL decay curves at 490 nm and 540 nm (Figure 3c) for both the pristine and ground samples were monitored. The lifetimes of the ground sample at both wavelengths are shorter than those of the pristine sample (for ground sample: 490 nm, τ_1_ = 1.03 ns, τ_2_ = 21.27 ns; 540 nm, τ_1_ = 1.19 ns, τ_2_ = 22.95 ns. for pristine sample: 490 nm, τ_1_ = 0.79 ns, τ_2_ = 24.29 ns; 540 nm, τ_1_ = 0.98 ns, τ_2_ = 31.47 ns). The spontaneously recovering luminescence switching in response to mechanical force demonstrates excellent reproducibility over multiple cycles with negligible variations in emission wavelength (Figure 3d).

To elucidate the origin of mechanical force‐responsive luminescence switching in (C_9_NH_20_)_6_[Pb_3_Br_12_], the crystal structure changes were first evaluated by PXRD (Figure 3e). The diffraction peak position exhibits almost no change after grinding. The relative intensity of some diffraction peaks alters, indicating the preferred orientation of crystals is eliminated with the anisotropic reduction in grain size. During secondary grinding, neither the diffraction peak positions nor the relative intensities show obvious changes. The Raman spectra of the pristine and ground samples were compared (Figure 3f). Peaks at 100–250 cm^−1^ are attributed to Pb–Br vibrations, and those in the range of 250–1500 cm^−1^ are assigned to C–H and N–H vibrations from the organic cations. No significant changes are observed in the Raman spectra of the ground sample compared to the pristine sample. The local structure changes were further investigated using solid‐state ^1^H and ^13^C NMR spectroscopy. In the ^13^C NMR spectrum (Figure S25, Supporting Information), the chemical shifts (δ) corresponding to C in different chemical environments are assigned. The chemical shifts and width in the ^13^C NMR spectra are highly consistent for the pristine and ground samples. However, the ^1^H NMR spectra exhibit some changes: the δ_H_ peaks at 4–5 ppm shift upfield, while those at 0–4 ppm shift slightly downfield compared to the pristine sample. Additionally, the δ_H_ peaks in the ground sample exhibit slight narrowing (Figure 3g). These observations indicate that grinding induces modifications in the local chemical environment of the organic cations.

**Figure 4:**
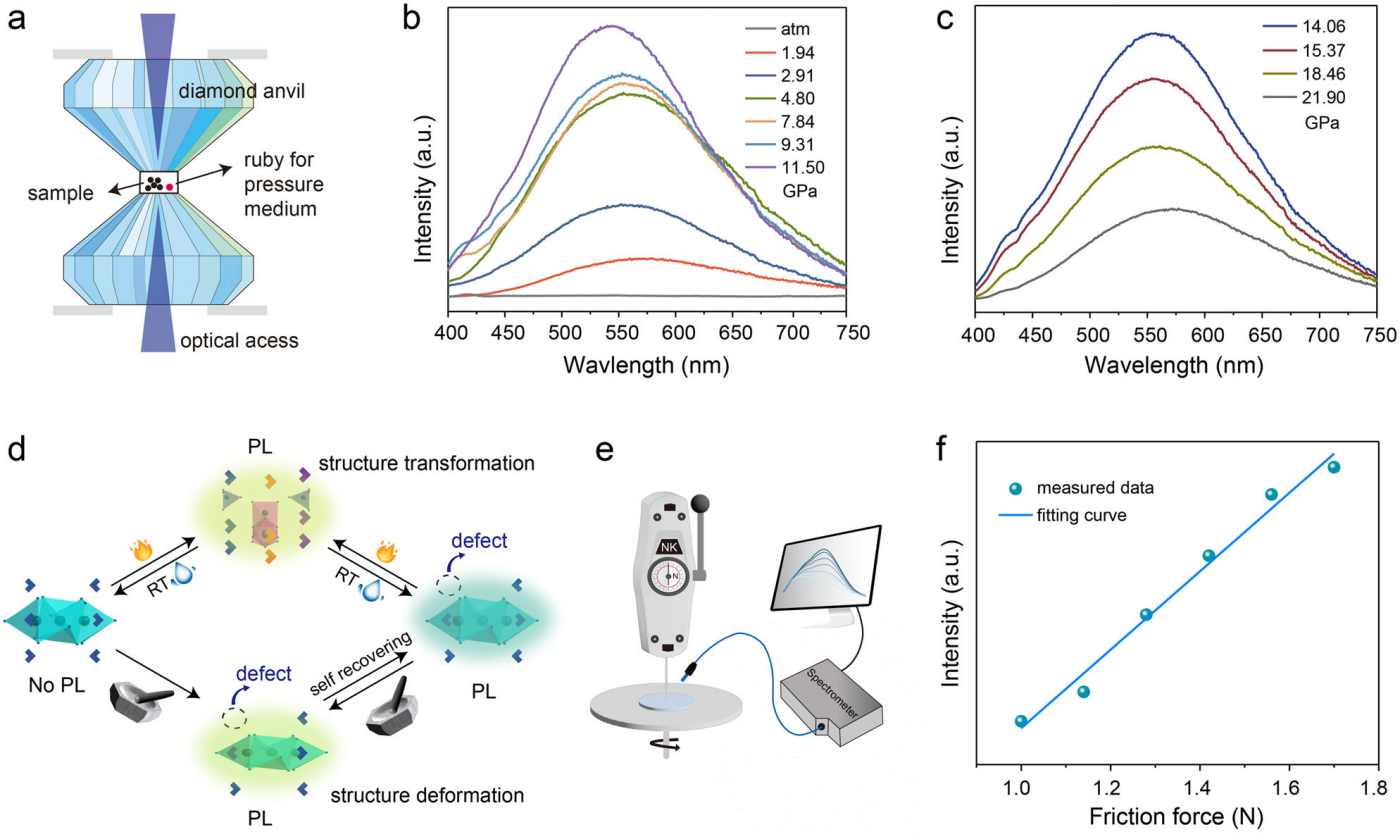
a) Schematic diagram of diamond anvil cell device to control the hydrostatic pressure of (C_9_NH_20_)_6_[Pb_3_Br_12_]. PL emission spectra of (C_9_NH_20_)_6_[Pb_3_Br_12_] in the pressure range of b) atm‐11.5 GPa and c) 14.06–21.90 GPa. d) Schematic illustration of the mechanism behind the stimuli‐responsive luminescence switching in (C_9_NH_20_)_6_[Pb_3_Br_12_]. e) Schematic diagram of the custom‐built test system. f) The luminescence intensity in response to forces of different magnitudes.

The pressure‐dependent luminescence properties of (C_9_NH_20_)_6_[Pb_3_Br_12_] were also investigated. **Figure** **4**a depicts the schematic diagram of the pressure control device with a diamond anvil cell. Due to the small amount of samples loaded into the sample holder, its green luminescence is barely discernible in the PL emission spectra at atmospheric pressure (Figure 4b). As the pressure increases to 1.94 GPa, a new emission peak emerges at 575 nm. With further pressure increase to 11.50 GPa, the intensity of this emission grows while its maximum continuously shifts to higher energies, reaching 555 nm at 6.62 GPa and 541 nm at 11.50 GPa. Beyond 11.50 GPa, the emission intensity decreases significantly (Figure 4c). The pressure‐dependent PL emission wavelength changes (Figure S26, Supporting Information) demonstrate that: the emission wavelength first redshifts due to the emergence of a new luminescent center, then blueshifts with further pressure increase. At even higher pressures, a slight redshift is observed. The new luminescence center is strongly correlated with the distortion of the [Pb_3_Br_12_]^6−^ motifs, which facilitates the radiative recombination of self‐trapped excitons by effectively lowering the activation energy required for de‐trapping these states.^[^ ^60^, ^61^ ^]^ Both isotropic compression under hydrostatic pressure and anisotropic force during grinding induce pronounced shifted emission, confirming the highly adjustable crystal lattice of (C_9_NH_20_)_6_[Pb_3_Br_12_]. The pressure‐dependent spectra and structure of non‐emissive (C_9_NH_20_)_6_[Pb_3_Br_12_] have been previously reported by Lü’s group.^[^ ^62^ ^]^ The green‐emitting (C_9_NH_20_)_6_[Pb_3_Br_12_] has same structure. Therefore, its lattice undergoes compression without amorphization or phase change. The blue‐shifted and enhanced emission during the initial pressurization stage can be ascribed to reduced electron‐vibrational coupling.^[^ ^63^, ^64^ ^]^ The weakened exciton‐phonon interaction, owing to the phonon hardening, leads to significant suppression of the phonon‐assisted non‐radiative pathway and consequently enhances emission intensity. With further compression under increasing pressure, the bandgap decreases and the wavefunction overlap between the self‐trapped emission state and the ground state increases, thereby causing a redshift and quenching of emission.

The normalized temperature‐dependent emission spectra at low temperatures (Figure S27, Supporting Information) reveal that the post‐grinding recovered sample, analogous to the green‐emitting sample after heating‐cooling‐exposure treatment, exhibits two emission centers with a slightly larger proportion of emission at 540 nm. At a controlled relative humidity of 22%, the mechano‐responsive yellow luminescence of (C_9_NH_20_)_6_[Pb_3_Br_12_] also transforms into green upon grinding cessation. Therefore, the green emission doesn't originate from moisture. It is deduced that mechanical grinding breaks the crystal into small particles with a high specific surface area. The weak interaction between C_9_NH_20_^+^ and [Pb_3_Br_12_]^6−^ in (C_9_NH_20_)_6_[Pb_3_Br_12_] leads to the generation of defects, which is consistent with the NMR results (Figure 3g). Simultaneously, grinding induces local structural deformation, which activates the trapped exciton emission at a long wavelength and results in the mechanical‐responsive luminescence switching in (C_9_NH_20_)_6_[Pb_3_Br_12_]. Unlike static pressure by a diamond anvil, dynamic mechanical force leads to a shorter emission wavelength for the activated trapped exciton emission. The variation in lifetimes of 490 and 540 nm for the pristine and ground samples (Figure 3c) illustrates the difference in radiative rates of defect‐trapped exciton emission and deformation‐related trapped exciton emission. The similar emission spectra of the grinding‐recovered and heating‐recovered samples can be attributed to the stabilization of defects. The mechanism behind the stimuli‐responsive luminescence switching in (C_9_NH_20_)_6_[Pb_3_Br_12_] was schematically illustrated (Figure 4d). (C_9_NH_20_)_6_Pb_3_Br_12_ consists of isolated [Pb_3_Br_12_]^6−^ cluster surrounded by C_9_NH_20_^+^ cations, with the C─H···Br bonds therein. Upon heating, the weak C─H···Br bonds break first, followed by destabilization and rearrangement of Pb─Br bonds, resulting in a structure transformation to (C_9_NH_20_)_9_(PbBr_4_)_2_[Pb_3_Br_11_] accompanied by the extraction of C_9_NH_20_Br. During cooling and humidity exposure, Br^−^ ion migration is accelerated, enhancing the reactivity between C_9_NH_20_Br with (C_9_NH_20_)_9_(PbBr_4_)_2_[Pb_3_Br_11_], which facilitates the recovery of the thermodynamically stable (C_9_NH_20_)_6_[Pb_3_Br_12_] structure. These structure transformations lead to heat/moisture ‐ responsive luminescence switching. Incomplete insertion of C_9_NH_20_Br during recovery introduces defects, altering the luminescence color of the recovered structure. When excess C_9_NH_20_Br is present, the recovered structure remains defect‐free and non‐luminescent as the bulk (C_9_NH_20_)_6_[Pb_3_Br_12_] single crystal. Grinding distorts the structure and introduces defects, leading to luminescence switching and then self‐recovering to the luminescence state resembling the defected (C_9_NH_20_)_6_[Pb_3_Br_12_] from heating‐cooling‐exposure.

A custom‐built dynamometry setup was employed to evaluate the relationship between applied force (f) and luminescent intensity (I) in (C_9_NH_20_)_6_[Pb_3_Br_12_] (Figure 4e; Video S2, Supporting Information). A (C_9_NH_20_)_6_[Pb_3_Br_12_] film was fabricated using a UV‐curable adhesive. The film was subjected to forces of varying magnitudes, revealing a strong linear correlation between f and I (Figure 4f; Figure S28, Supporting Information), highlighting the potential of (C_9_NH_20_)_6_[Pb_3_Br_12_] for quantitative visualization of dynamic forces.

### 2.4 Anti‐Counterfeiting and Force Sensing Applications

**Figure 5:**
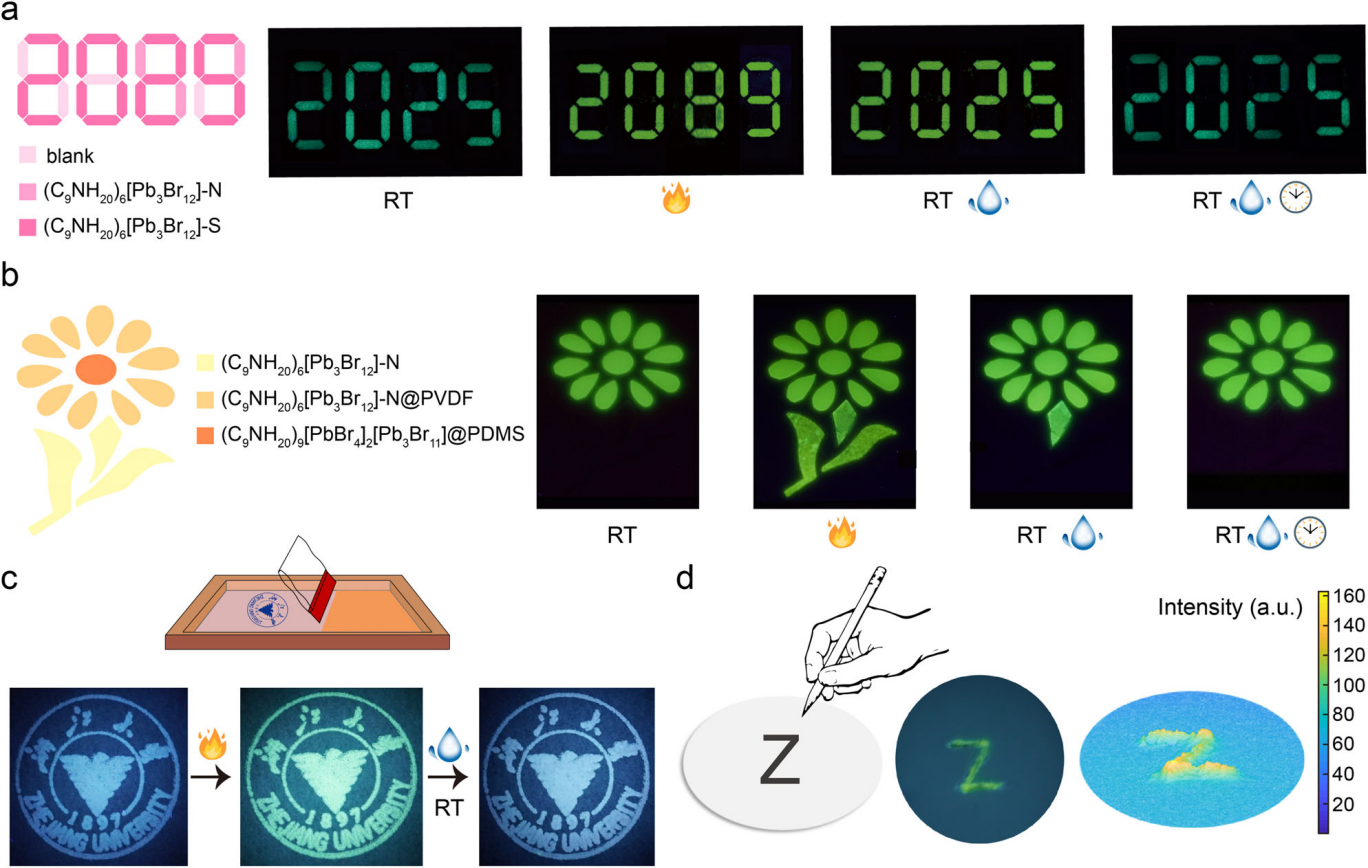
a) Optical anti‐counterfeiting patterns created using the “8888” module. The slots of module are selectively filled with stoichiometric (C_9_NH_20_)_6_[Pb_3_Br_12_] (abbreviated as (C_9_NH_20_)_6_[Pb_3_Br_12_]‐S) and nonstoichiometric (C_9_NH_20_)_6_[Pb_3_Br_12_] (abbreviated as (C_9_NH_20_)_6_[Pb_3_Br_12_]‐N). b) Dynamic anti‐counterfeiting with flower patterns created using nonstoichiometric (C_9_NH_20_)_6_[Pb_3_Br_12_], nonstoichiometric (C_9_NH_20_)_6_[Pb_3_Br_12_]@PVDF (abbreviated as (C_9_NH_20_)_6_[Pb_3_Br_12_]‐N@ PVDF), and (C_9_NH_20_)_6_[PbBr_4_]_2_[Pb_3_Br_11_]@PDMS. c) Schematic of screen‐printing process using (C_9_NH_20_)_6_[Pb_3_Br_12_] ink, and luminescence of the printed Zhejiang University logo during a heating‐cooling‐exposure cycle. d) Schematic of handwriting using (C_9_NH_20_)_6_[Pb_3_Br_12_] film as “paper”, and force visualization for the handwritten letter “Z”. All the photographs were taken under 365 nm excitation.

Given its unique stimuli‐responsive fluorescence switching and good reversibility, the potential application of (C_9_NH_20_)_6_[Pb_3_Br_12_] in high‐security anti‐counterfeiting was evaluated. In a proof‐of‐concept demonstration, stoichiometric and nonstoichiometric (C_9_NH_20_)_6_[Pb_3_Br_12_] were selectively filled into the groove of 8‐shaped modules (**Figure** **5**a). Under ambient conditions, the 8‐shaped modules remain inconspicuous. However, under 365 nm UV excitation, the parts filled with stoichiometric (C_9_NH_20_)_6_[Pb_3_Br_12_] emit green light, presenting a pattern of “2025”. When heated to 130 °C, both types of (C_9_NH_20_)_6_[Pb_3_Br_12_] transform into (C_9_NH_20_)_9_[PbBr_4_]_2_[Pb_3_Br_11_], and the pattern changes to “2089” with yellow luminescence appears under UV excitation. As previously described, the heat‐converted structure from nonstoichiometric (C_9_NH_20_)_6_[Pb_3_Br_12_] contains surplus C_9_NH_20_Br, which accelerates its recovery due to the high hygroscopicity of C_9_NH_20_Br. With prolonged exposure, the pattern of “2089” with yellow luminescence gradually changes to “2025” with yellow luminescence. This dual heat and moisture‐responsive optical anti‐counterfeiting model provides multicolor (including none‐emissive, yellow, and green states—exceeding two states) luminescence switching, offering superior security over conventional single‐stimulus, two‐state systems.

Leveraging the moisture‐dependent crystal transformation and luminescence switching, another high‐security anti‐counterfeiting model was further devised (Figure 5b). Nonstoichiometric (C_9_NH_20_)_6_[Pb_3_Br_12_], nonstoichiometric (C_9_NH_20_)_6_[Pb_3_Br_12_]@PVDF, and (C_9_NH_20_)_9_[PbBr_4_]_2_[Pb_3_Br_11_]@PDMS were selectively filled into the leaf, stem, and petal part of a flower pattern, respectively. The PDMS‐encapsulated (C_9_NH_20_)_6_[Pb_3_Br_12_] requires high‐temperature solidification, transforming it into (C_9_NH_20_)_9_[PbBr_4_]_2_[Pb_3_Br_11_] with yellow luminescence. The PDMS encapsulation prevents moisture ingress, ensuring the stability of (C_9_NH_20_)_9_[PbBr_4_]_2_[Pb_3_Br_11_]. First, under 365 nm UV excitation, the petal part emits yellow light. Second, at 130 °C, the leaf, stem, and petal parts all show yellow luminescence. Third, after removing the heating source, the yellow luminescence decays at varying rates. After 10 min, only the stem and petal parts remain yellow luminescence. With continuous exposure, the optical anti‐counterfeiting model is reset, with the petal part solely presenting yellow luminescence. This heat‐ and exposure‐time‐dependent luminescence behavior, governed by crystal transformations, enables sophisticated time‐dependent dynamic anti‐counterfeiting applications.

In addition to film fabrication through drop‐casting, (C_9_NH_20_)_6_[Pb_3_Br_12_] can also be precisely patterned. Screen printing, a widely adopted technique for depositing inks onto various surfaces, was used to create tightly packed patterns on filter paper. Polyvinylpyrrolidone (PVP) was added to increase the viscosity of (C_9_NH_20_)_6_[Pb_3_Br_12_] ink. The logo of Zhejiang University was printed using (C_9_NH_20_)_6_[Pb_3_Br_12_] ink (Figure 5c). Under 365 nm UV excitation, the printed logo shows blue emission from PVP. Upon heating, the luminescence changes to homogeneous yellow, which reverts to blue after cooling and environmental exposure. The screen‐printed pattern maintains the compound's heat‐ and moisture‐responsive structure transformations and luminescence switching properties, demonstrating its potential for wider practical anti‐counterfeiting applications.

The unique self‐recovering mechanical‐responsive luminescence switching of (C_9_NH_20_)_6_[Pb_3_Br_12_] also enables its use as a platform for analyzing handwriting habits (Figure 5d). The (C_9_NH_20_)_6_[Pb_3_Br_12_] film was fabricated by mixing the powder with a UV curing adhesive. When scratched with a ceramic spatula, the film's luminescence changes to yellow under UV excitation, visualizing a handwritten character “Z”. The mechano‐induced luminescence intensity variation along handwritten trajectories can be quantified through grayscale analysis of optical images. This capability allows for the analysis of handwriting characteristics, with applications in electronic signature and forgery prevention. Due to the self‐recovery property, the (C_9_NH_20_)_6_[Pb_3_Br_12_] paper is rewritable. Under 365 nm UV light, the luminescent line created by ceramic scratching fades over time, and additional luminescent line can be repeatedly inscribed (Figure S29, Supporting Information).

## 3 Conclusion

In summary, we have discovered a multimodal stimuli‐responsive luminescence with tunable luminescence color in (C_9_NH_20_)_6_[Pb_3_Br_12_] for advanced anti‐counterfeiting and handwriting recognition applications. The luminescence switching during heating‐cooling and moisture exposure cycles is driven by crystal structure transformation accompanied by the extraction/insertion of C_9_NH_20_Br. The reactivity of C_9_NH_20_Br during insertion depends on the moisture level and determines the recovery rate of the heat‐converted structure. During this process, incomplete C_9_NH_20_Br insertion creates defects that generate green luminescence in recovered structures, whereas complete insertion in the presence of excess C_9_NH_20_Br yields non‐emissive, defect‐free (C_9_NH_20_)_6_[Pb_3_Br_12_]. In addition, (C_9_NH_20_)_6_[Pb_3_Br_12_] displays self‐recovering luminescence switching in response to mechanical force. Structure analysis reveals no structure changes. Luminescence studies at low temperatures and high pressures illustrate that structure deformation‐induced trapped exciton emission leads to the increased low‐energy component in the emission spectrum and luminescence switching. A linear correlation exists between the magnitude of applied mechanical force and the intensity of luminescence switching. Based on the reversible temperature/moisture/mechanical force‐responsive luminescence switching, as well as tunable luminescence, we successfully demonstrate the application of (C_9_NH_20_)_6_[Pb_3_Br_12_] in time‐dependent high‐security anti‐counterfeiting and handwriting recognition. The findings introduce a new paradigm for stimuli‐responsive luminescent OMH materials and their applications while elucidating the underlying mechanisms.

## Conflict of Interest

The authors declare no conflict of interest.

## Supporting information



Supporting Information

Supplemental Video 1

Supplemental Video 2

## Data Availability

The data that support the findings of this study are available from the corresponding author upon reasonable request.
