# Supplementary material for: Stimuli‐Responsive and Defect‐Regulated Luminescent Organic Metal Halide for High‐Security Anti‐Counterfeiting and Force Sensing
Source: Adv Sci (Weinh). 2025 Jul 12;12(38):e10163. doi: 10.1002/advs.202510163 (PMC12520570; doi:10.1002/advs.202510163)
Supplement: Supplementary file 1 — Supporting Information [file ADVS-12-e10163-s002.pdf]

ADVANCED SCIENCE
Open Access

Supporting Information

for *Adv. Sci.*, DOI 10.1002/advs.202510163

Stimuli-Responsive and Defect-Regulated Luminescent Organic Metal Halide for High-Security Anti-Counterfeiting and Force Sensing

Chunyan Jiang, Jing Yan, Rongkai Du, Yang Li, Mingmei Wu, Beibei Xu* and Jianrong Qiu*

Supporting Information

Stimuli-Responsive and Defect-Regulated Luminescent Organic Metal Halide for High-Security Anti-Counterfeiting and Force Sensing

Chunyan Jiang, Jing Yan, Rongkai Du, Yang Li, Mingmei Wu, Beibei Xu*, and Jianrong Qiu*

Experimental Section

Chemicals: $PbBr_2$ (p-oled, 99.99%), *1*-butyl-*1*-methylpyrrolidinium bromide ($C_9NH_{20}Br$, Sigma, 99.0%), DMF (Sigma, 99.9%), acetonitrile (Macklin, 99.9%), acetone (Guangzhou Chemical Reagent Factory, 99.5%), polyvinyl pyrrolidone (PVP, Aladdin, 40000), Polydimethylsiloxane (PDMS, SYLGARD, DC184), UV curable adhesive (Norland, NOA61), and polyvinylidene fluoride (PVDF, Aladdin, 400000) were used as starting materials, all reagents were used without further purification.

Synthesis of $(C_9NH_{20})_6Pb_3Br_{12}$ single crystal: A 1:4 ratio of $PbBr_2$ (0.27 mmol) and $C_9NH_{20}Br$ (1.1 mmol) was weighed and dissolved in 3 mL DMF. After stirring at 60 °C for 10 minutes, the solution became transparent. Following filtration with a syringe filter, the solution was left to sit undisturbed. Bulk crystals of $(C_9NH_{20})_6Pb_3Br_{12}$ were obtained after one day. The resulting colorless crystals were washed with acetone and dried under vacuum.

Synthesis of nonstoichiometric $(C_9NH_{20})_6Pb_3Br_{12}$ powder: A 1:4 ratio of $PbBr_2$ (0.27 mmol) and $C_9NH_{20}Br$ (1.1 mmol) was weighed and dissolved in 4 mL acetonitrile. The solution was sufficiently stirred until transparent, and then placed on a heating plate at a temperature of 80 °C. Upon complete evaporation of the acetonitrile, nonstoichiometric $(C_9NH_{20})_9[PbBr_4]_2[Pb_3Br_{11}]$ powder was obtained. After cooling and storage, this powder transformed into $(C_9NH_{20})_6Pb_3Br_{12}$ powder.

Synthesis of nonstoichiometric $(C_9NH_{20})_6Pb_3Br_{12}$@PVDF: 0.27 mmol of $PbBr_2$, 1.1 mmol of $C_9NH_{20}Br$, and 0.2 g of PVDF were weighed and dissolved in 3 mL DMF. The solution was stirred for a duration of 30 minutes to ensure complete dissolution. The transparent solution was carefully dropped into the template and subsequently transferred into a heating oven. The heating protocol involved maintaining a temperature of 60 °C for 6 hours, followed by an increase to 100 °C for an additional 12 hours. The nonstoichiometric $(C_9NH_{20})_6Pb_3Br_{12}$@PVDF was obtained after cooling and storage.

Synthesis of $(C_9NH_{20})_9[PbBr_4]_2[Pb_3Br_{11}]$@PDMS: The bulk $(C_9NH_{20})_6Pb_3Br_{12}$ single crystals were ground into a fine powder. PDMS glue composed of A and B component in a 1:10 ratio was mixed with the $(C_9NH_{20})_6Pb_3Br_{12}$ powder. The mixture was dropped into a template, followed by heating at 130 °C for 12 hours. All procedures were conducted within a glove box.

Screen-printed patterned $(C_9NH_{20})_6Pb_3Br_{12}$ film: A 1:4 ratio of $PbBr_2$ (0.27 mmol) and $C_9NH_{20}Br$ (1.1 mmol) was weighed and dissolved in 3 mL DMF. To improve the adhesion and durability of the subsequent handwriting patterns onto filter paper, 0.3 g of PVP powder was added into the solution. After stirring for 30 minutes to ensure complete dissolution, the solution was filtered through a syringe filter to remove any undissolved particles or impurities.

Subsequently, the precursor solution was employed for screen printing, with filter paper serving as the substrate. Following screen printing, the pattern-bearing filter paper was transferred to a heating oven with a temperature of 80 °C for 12 hours. The patterned $(C_9NH_{20})_6Pb_3Br_{12}$ film was obtained after cooling and storage.

Characterization: Single crystal diffraction data was collected on a Bruker D8 Venture diffractometer equipped with a liquid metal target (Ga-Kα radiation, λ = 1.34138 Å) at 150 K. The crystal structure was resolved and refined using direct methods with SHELX. Powder diffraction data (PXRD) was collected on a Bruker D8 Advance diffractometer with Cu-Kα radiation (λ = 1.54178 Å) at 40 kV and 40 mA. Temperature-dependent PXRD measurements were performed on Brucker D8 Advance diffractometer at 35 kV and 35 mA. Thermogravimetric (TG) analysis was carried out under N2 flow of 20 mL/min at a rate of 10 °C/min using a NetzschTG209F3 TGA instrument. Differential scanning calorimetry (DSC) analysis was performed under N2 flow of 20 mL/min at a rate of 10 °C/min using a NetzschTG209F3 DSC instrument. UV-visible transmittance and diffusion reflection spectra were recorded using a Cary 5000 UV–vis–near-IR spectrophotometer by using $BaSO_4$ powder as the standard reference. Raman spectra were recorded using the RENISHAW InVia-Reflex Micro-Confocal Raman spectrometer with an incident laser wavelength of 532 nm. X-ray photoelectron spectra (XPS) were measured using the Thermo Fisher/ESCALAB Oxi instrument. Nuclear magnetic resonance (NMR) spectra were recorded on a Bruker Avance Av 400MHz NMR instrument. Steady-state and time-resolved photoluminescence (PL) spectra were measured on a FLS1000 spectrometer (Edinburgh Instruments), 450 W Xenon lamp and 365 nm picoseconds pulsed diode laser with a repetition rate of 10 MHz (100 ns) were used as excitation source, respectively. Temperature dependent PL spectra were acquired with the same spectrometer, where temperature was precisely controlled using an Oxford cryogenic system within the range of 77-500 K. In-situ pressure-dependent PL spectra were also captured using the FLS1000 spectrometer. High pressures were generated using a symmetric diamond anvil cell, and a ruby was employed to determine the actual pressure through the standard ruby fluorescent technique. Photoluminescence quantum yield was measured on an absolute quantum efficiency testing system (Hamamatsu, C9920). The mechano-chromic PL spectra were acquired using a self-built equipment comprising a ZQ-32 pressure tester (Dongguan ZHIQU Precision Instrument Co. Ltd), a TC-218 spin-coater (Shenyang SYSILE machinery factory), and a CH-QE90 fiber optic spectrometer (Ocean Optics). The 3D stress visualization image was drawn using MATLAB software.

Table S1. Crystallographic data and refinement details for $(C_9H_{20}N)_6Pb_3Br_{12}$.

Compound	$(C_9H_{20}N)_6Pb_3Br_{12}$ (CCDC 2467631)
Empirical formula	C54H120N6Pb3Br12
Formula Mass	2434.04
T/K	150 K
Wavelength	1.34138 Å
Crystal system	Trigonal
Space group	R -3
a (Å)	17.331(2)
b (Å)	17.331(2)
c (Å)	21.787(2)
α (°)	90°
β (°)	90°
γ (°)	120°
Volume	5667.0(15) $Å^3$
Z	3
Density (calculated)	2.140 g/m^3
Absorption coefficient	13.694 mm^{-1}
F(000)	3456.0
Crystal size	0.005 × 0.003 × 0.002 mm^3
Theta range for data collection	3.110 to 59.232°
Index ranges	-21<=h<=20, -21<=k<=21, -27<=l<=27
Reflections collected	17343
Independent reflections	9742 [R(int) = 0.0705]
Completeness to theta = 25.242°	0.996
Refinement method	Full-matrix least-squares on F^2
Data / restraints / parameters	2775 / 0 / 116
Goodness-of-fit on F^2	1.080
Final R indices [I>2sigma(I)]	R1 = 0.0297, wR2 = 0.0693
R indices (all data)	R1 = 0.0393, wR2 = 0.0723
Largest diff. peak and hole	0.798 and -1.363 e $Å^{-3}$

Table S2. Crystallographic data and refinement details for $(C_9NH_{20})_2Pb_2Br_6$.

Compound	$(C_9NH_{20})_2Pb_2Br_6$ (CCDC 2467480)
Empirical formula	C18H40Br6N2Pb2
Formula Mass	1178.32
T/K	150 K
Wavelength	0.71073 Å
Crystal system	Orthorhombic
Space group	I m a 2
a (Å)	9.3947(8)
b (Å)	19.3498(17)
c (Å)	8.0508(7)
α (°)	90°
β (°)	90°
γ (°)	90°
Volume	1463.5(2) $Å^3$
Z	2
Density (calculated)	2.674 g/m^3
Absorption coefficient	19.688 mm^{-1}
F(000)	1072.0
Crystal size	0.01 × 0.006 × 0.003 mm^3
Theta range for data collection	2.105 to 27.090°
Index ranges	-11<=h<=12, -24<=k<=24, -10<=l<=10
Reflections collected	16884
Independent reflections	9954 [R(int) = 0.0495]
Completeness to theta = 25.242°	1.000
Refinement method	Full-matrix least-squares on F^2
Data / restraints / parameters	1708 / 84 / 117
Goodness-of-fit on F^2	1.067
Final R indices [I>2sigma(I)]	R1 = 0.0432, wR2 = 0.11043
R indices (all data)	R1 = 0.0438, wR2 = 0.1113
Largest diff. peak and hole	0.462 and -1.296 e $Å^{-3}$

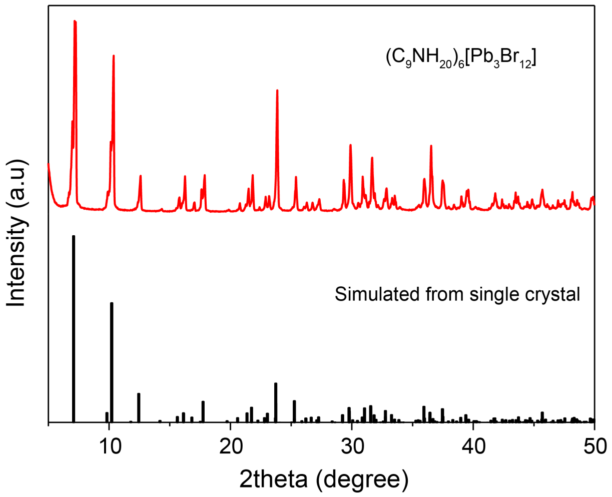

Figure S1. PXRD pattern of $(C_9NH_{20})_6[Pb_3Br_{12}]$ single crystals.

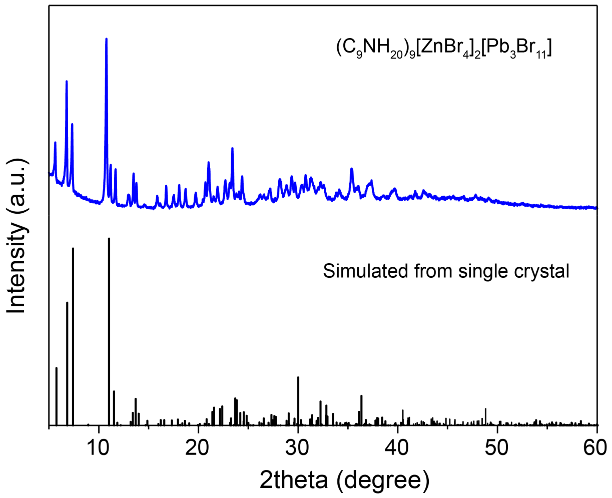

Figure S2. PXRD pattern of $(C_9NH_{20})_9[ZnBr_4]_2[Pb_3Br_{12}]$ single crystals.

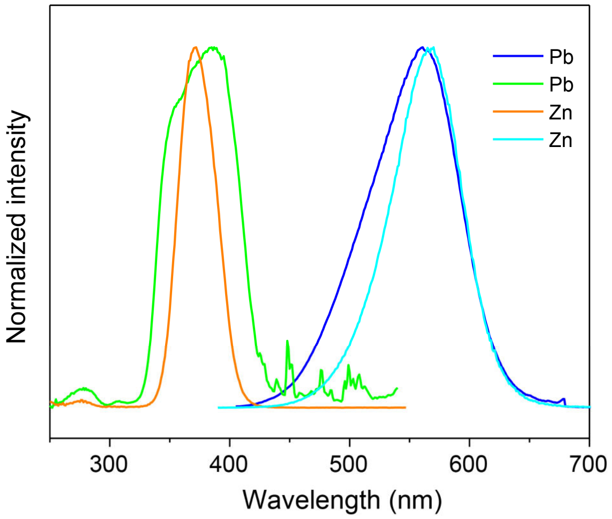

Figure S3. PL excitation/emission spectra of $(C_9NH_{20})_9[ZnBr_4]_2[Pb_3Br_{12}]$ and $(C_9NH_{20})_9[PbBr_4]_2[Pb_3Br_{12}]$.

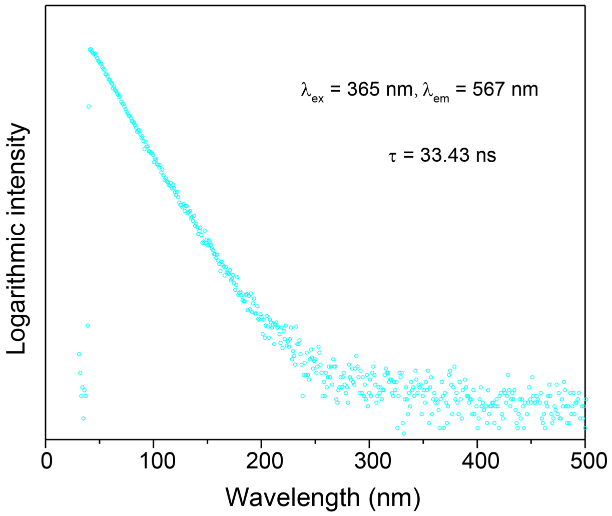

Figure S4. Time-resolved PL spectra of $(C_9NH_{20})_9[ZnBr_4]_2[Pb_3Br_{12}]$.

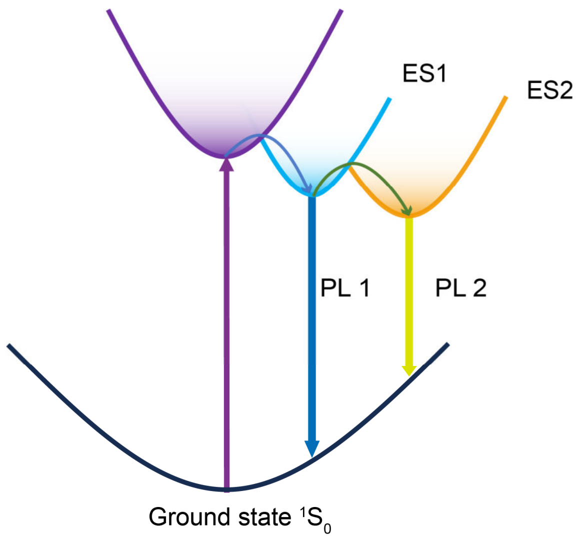

Figure S5. Schematic diagram of the photophysical process of inorganic $[Pb_3Br_{11}]^{5-}$ moiety.

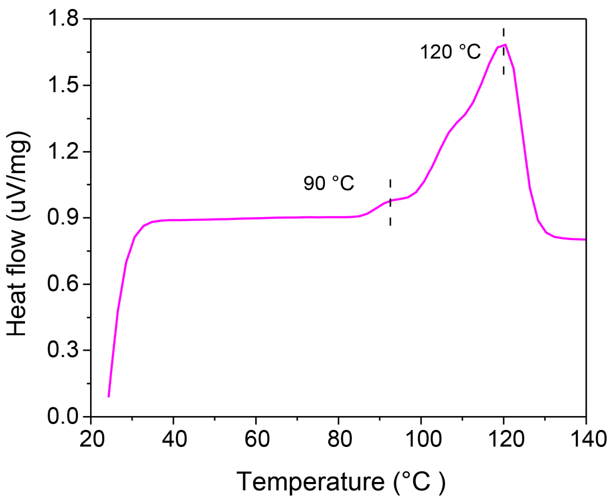

Figure S6. DSC curve of $(C_9NH_{20})_6[Pb_3Br_{12}]$ at heating.

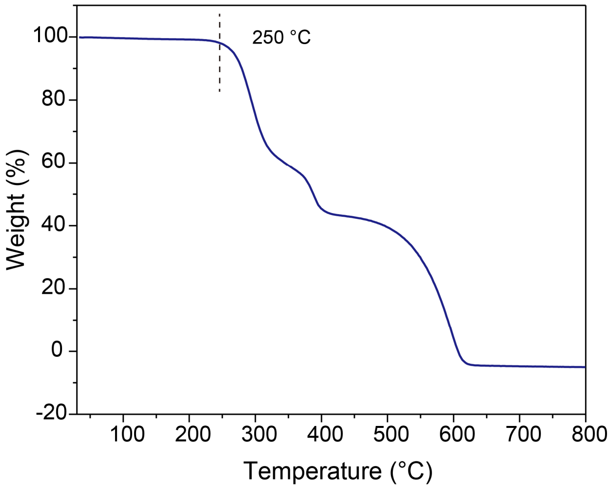

Figure S7. TG curve of $(C_9NH_{20})_6[Pb_3Br_{12}]$.

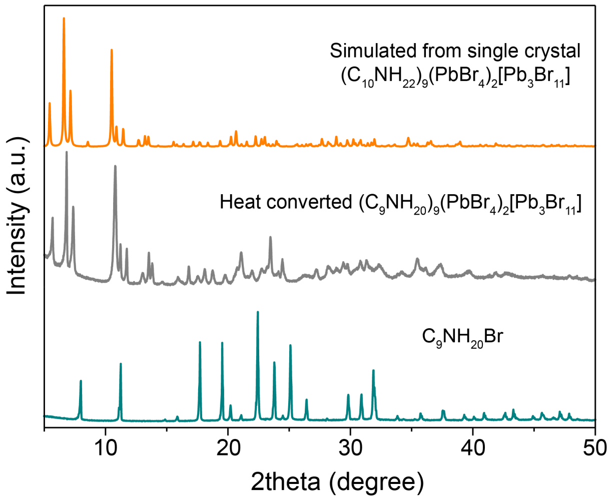

Figure S8. Simulated XRD pattern from single crystal $(C_{10}NH_{22})_9[PbBr_4]_2[Pb_3Br_{11}]$ and experiment XRD patterns of the heat-converted $(C_9NH_{20})_9[PbBr_4]_2[Pb_3Br_{11}]$ and $C_9NH_{20}Br$.

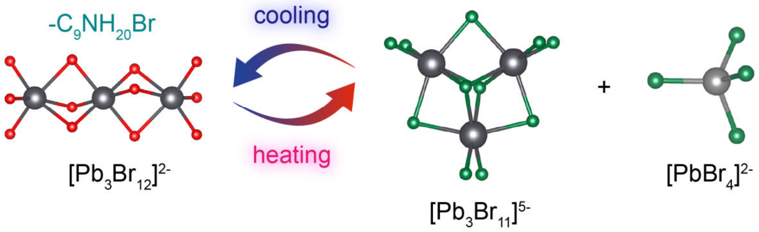

Figure S9. Pb coordination changes in the crystal structure transition during heating-cooling-exposure process.

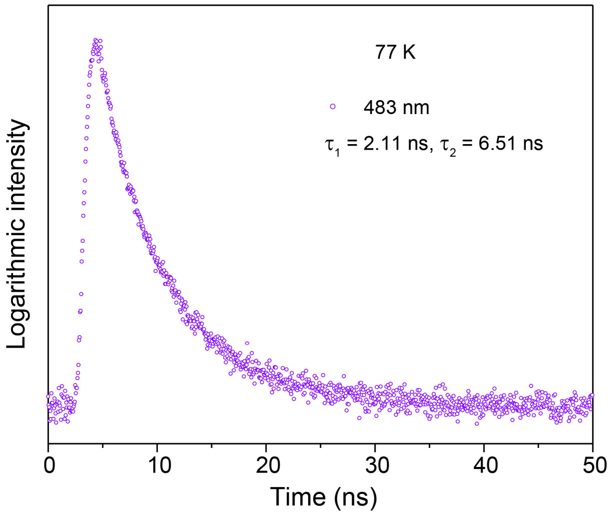

Figure S10. Time-resolved PL spectra of green-emitting $(C_9NH_{20})_6[Pb_3Br_{12}]$ at 77K (λ_{ex} = 365 nm).

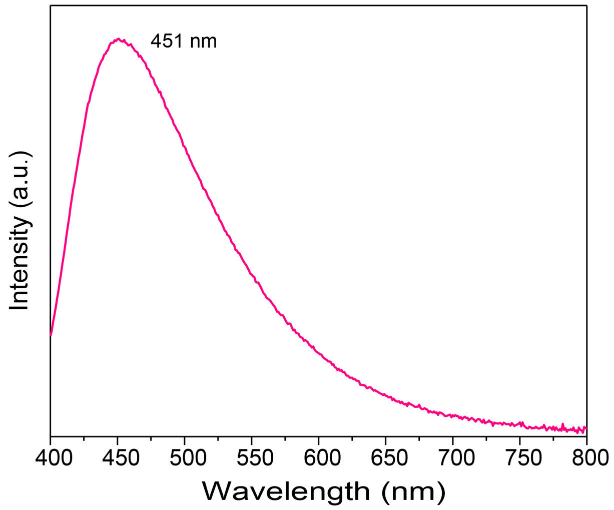

Figure S11. PL emission spectra of $C_9NH_{20}Br$ at RT (λ_{ex} = 365 nm).

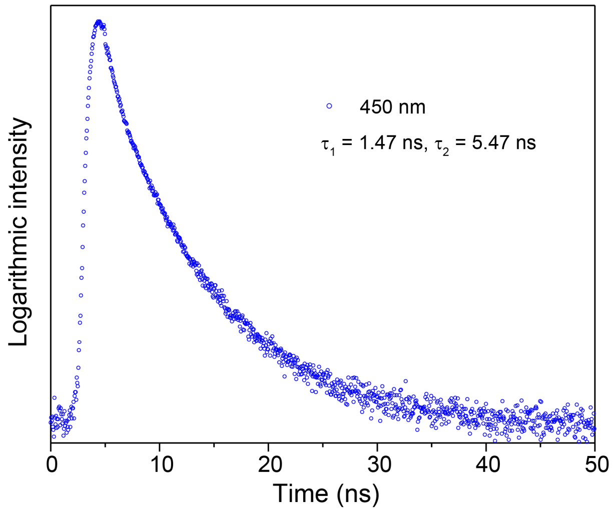

Figure S12. Time-resolved PL spectra of $C_9NH_{20}Br$ at RT (λ_{ex} = 365 nm).

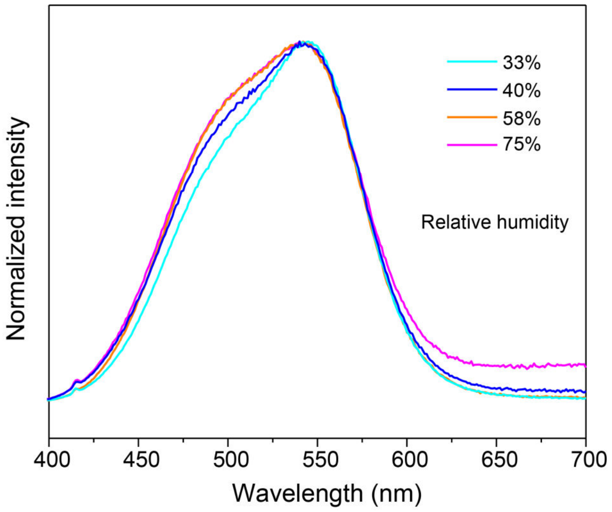

Figure S13. Normalized emission spectra of stoichiometric $(C_9NH_{20})_6[Pb_3Br_{12}]$ samples recovered from different relative humidity conditions.

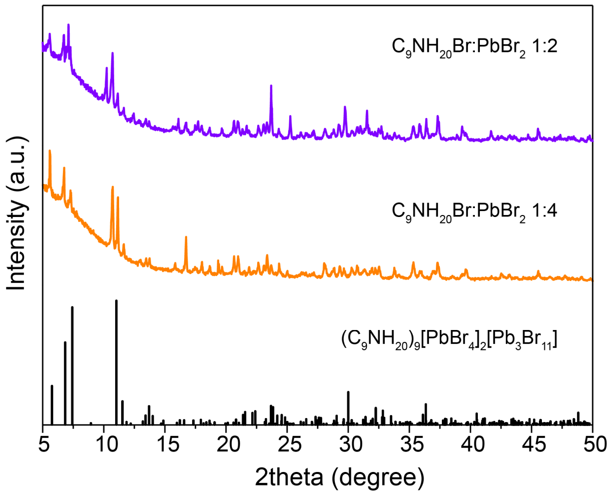

Figure S14. PXRD patterns of solution evaporation-synthesized samples with different ratios of $C_9NH_{20}Br$ and $PbBr_2$.

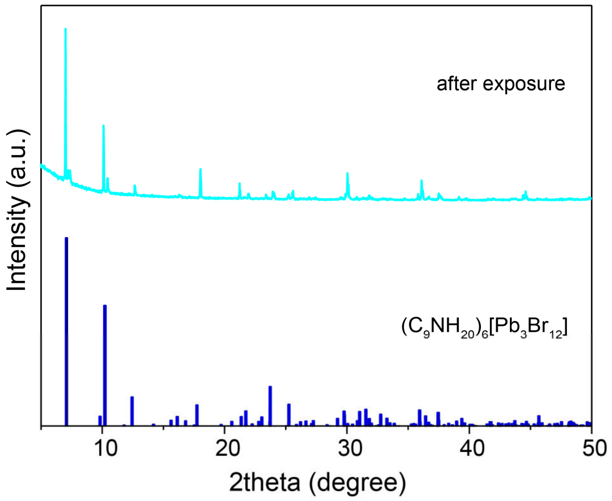

Figure S15. PXRD pattern of solution evaporation-synthesized $(C_9NH_{20})_9[PbBr_4]_2[Pb_3Br_{11}]$ after cooling-exposure.

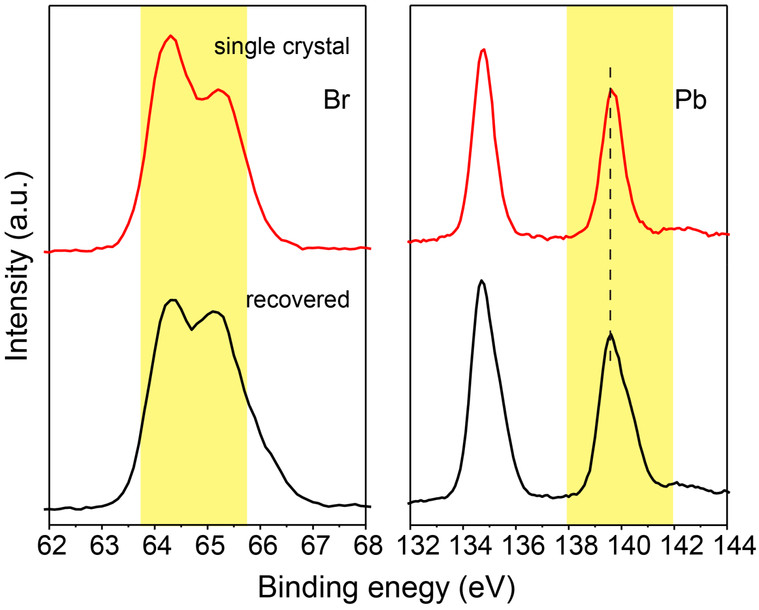

Figure S16. XPS spectra of $(C_9NH_{20})_6[Pb_3Br_{12}]$ single crystal and recovered $(C_9NH_{20})_6[Pb_3Br_{12}]$ powder.

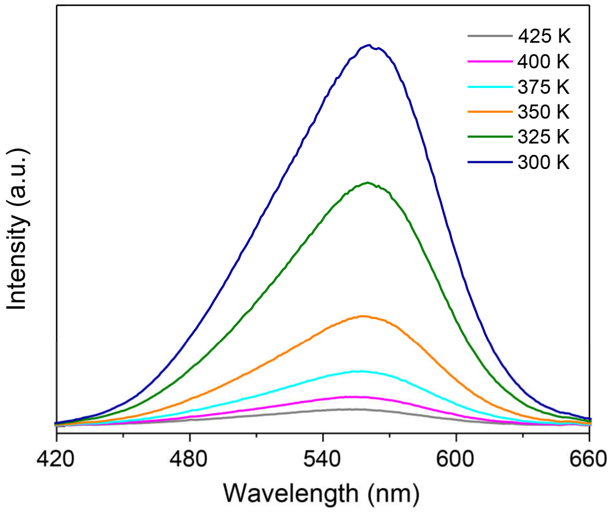

Figure S17. Temperature dependent PL emission spectra of the heat-converted $(C_9NH_{20})_9[PbBr_4]_2[Pb_3Br_{12}]$ at cooling process.

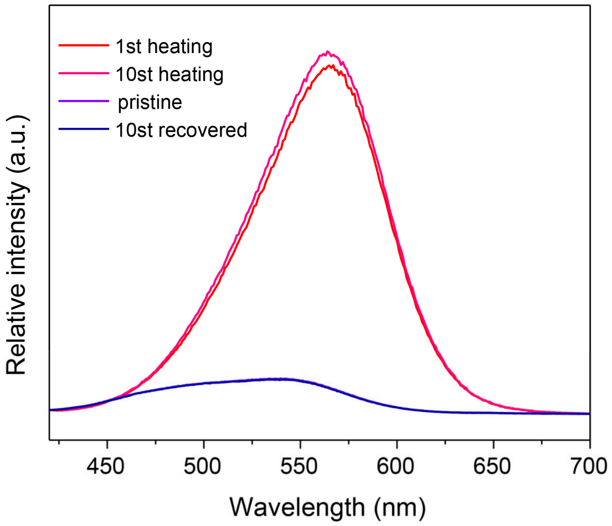

Figure S18. PL emission spectra of stoichiometric $(C_9NH_{20})_6[Pb_3Br_{12}]$ and heat-converted $(C_9NH_{20})_9[PbBr_4]_2[Pb_3Br_{11}]$, before and after 10 cycles of heating-cooling-exposure.

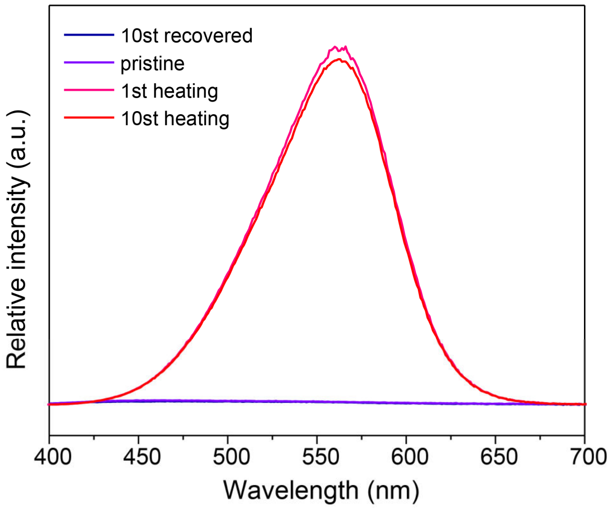

Figure S19. PL emission spectra of non-stoichiometric $(C_9NH_{20})_6[Pb_3Br_{12}]$ and heat-converted $(C_9NH_{20})_9[PbBr_4]_2[Pb_3Br_{11}]$, before and after 10 cycles of heating-cooling-exposure.

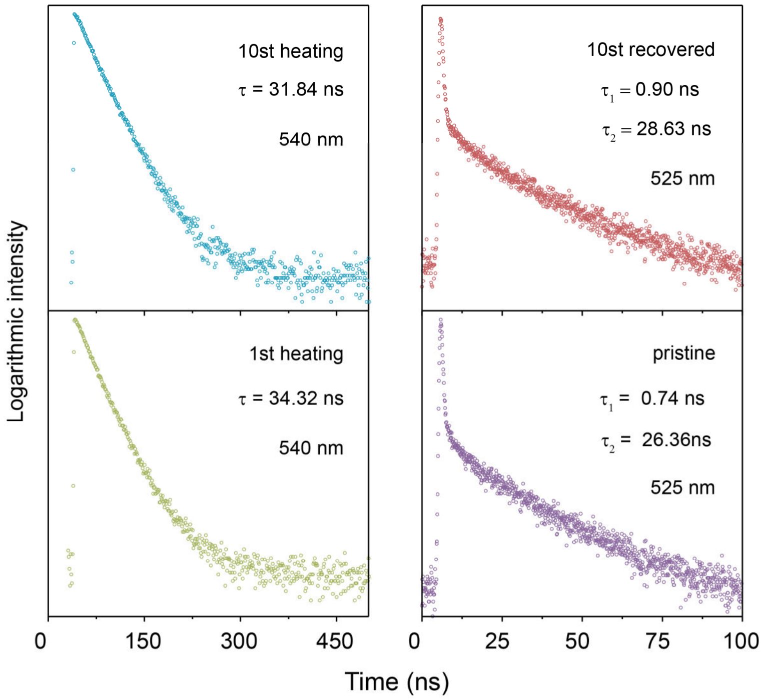

Figure S20. PL decay curves of stoichiometric $(C_9NH_{20})_6[Pb_3Br_{12}]$ and heat-converted $(C_9NH_{20})_9[PbBr_4]_2[Pb_3Br_{11}]$, before and after 10 cycles of heating-cooling-exposure.

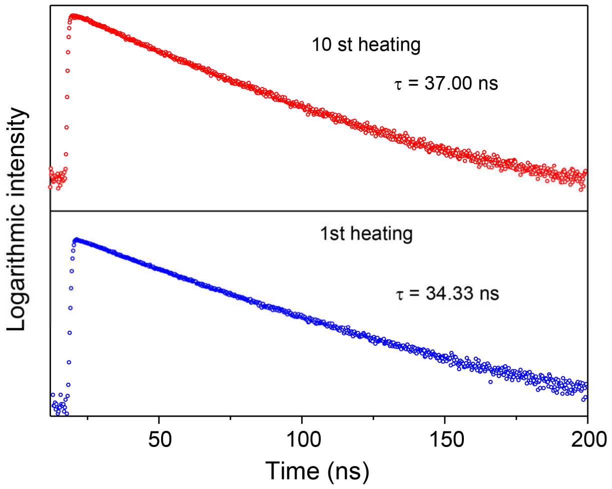

Figure S21. Time-resolved PL spectra of heat-converted non-stoichiometric $(C_9NH_{20})_6[Pb_3Br_{12}]$ before and after 10 cycles of heat-cooling-exposure.

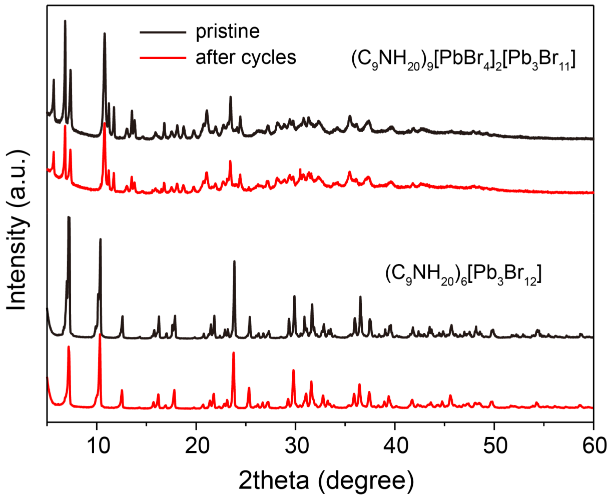

Figure S22. PXRD patterns of stoichiometric $(C_9NH_{20})_6[Pb_3Br_{12}]$ and heat-converted $(C_9NH_{20})_9[PbBr_4]_2[Pb_3Br_{11}]$, before and after 10 cycles of heat-cooling-exposure.

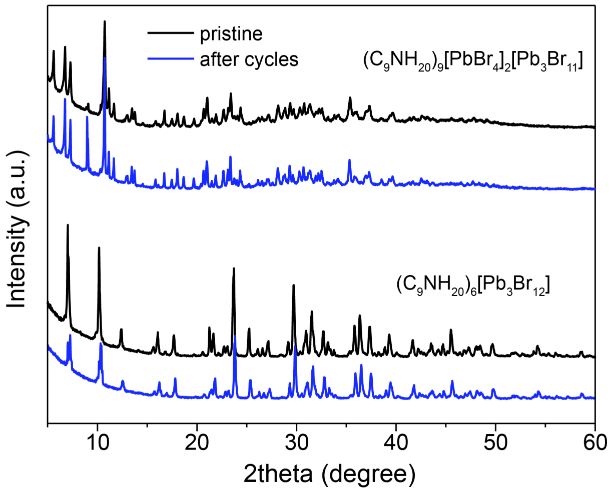

Figure S23. PXRD patterns of non-stoichiometric $(C_9NH_{20})_6[Pb_3Br_{12}]$ and heat-converted $(C_9NH_{20})_9[PbBr_4]_2[Pb_3Br_{11}]$, before and after 10 cycles of heat-cooling-exposure.

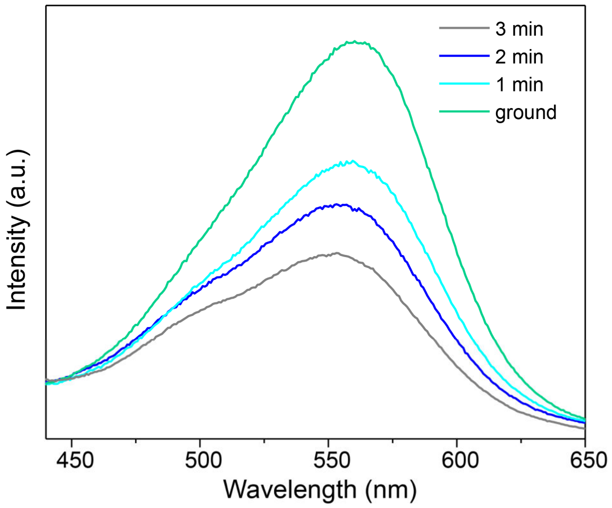

Figure S24. PL emission spectra of $(C_9NH_{20})_6[Pb_3Br_{12}]$ in its ground state and after grinding for various durations (λ_{ex} = 365 nm).

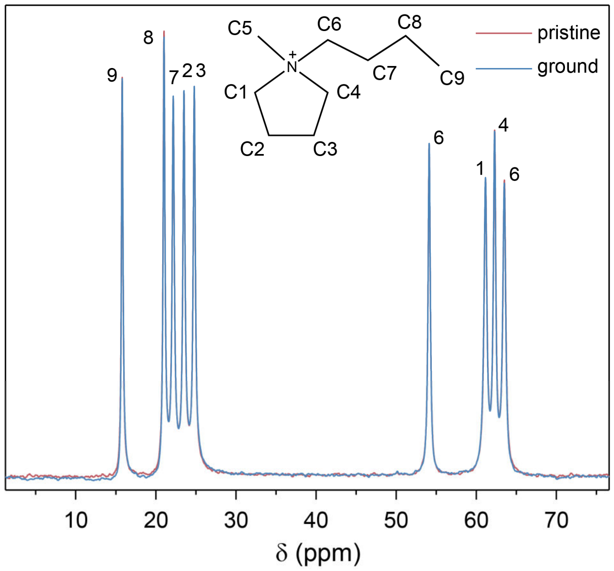

Figure S25. NMR C[13] spectra of the pristine and ground $(C_9NH_{20})_6[Pb_3Br_{12}]$, C atoms of $(C_9NH_{20})_6[Pb_3Br_{12}]$ corresponding to their NMR C[13] signal are assigned.

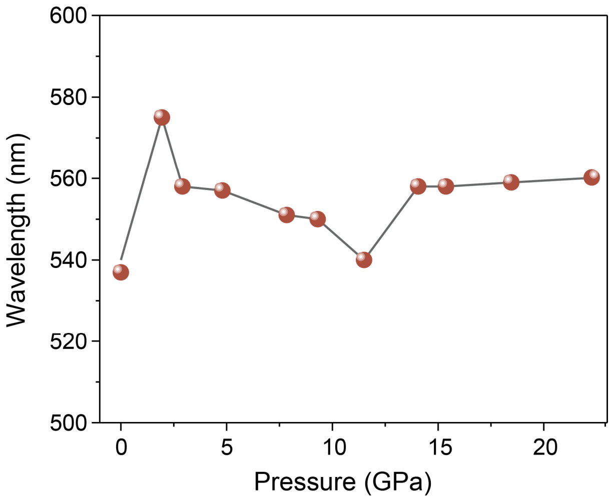

Figure S26. Emission wavelength variation of $(C_9NH_{20})_6[Pb_3Br_{12}]$ under different pressures.

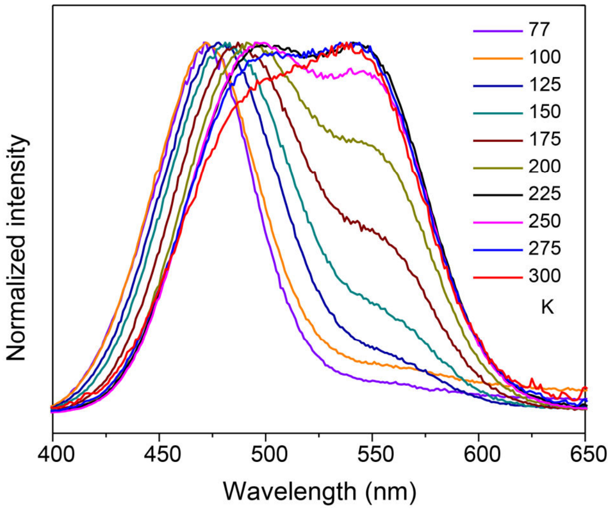

Figure S27. Temperature-dependent normalized PL emission spectra of $(C_9NH_{20})_6[Pb_3Br_{12}]$ after post-grinding recovery.

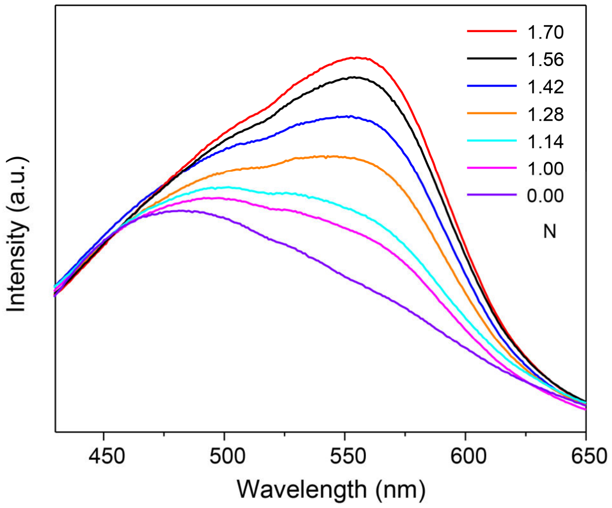

Figure S28. PL emission spectra of $(C_9NH_{20})_6[Pb_3Br_{12}]$ film under different mechanical forces.

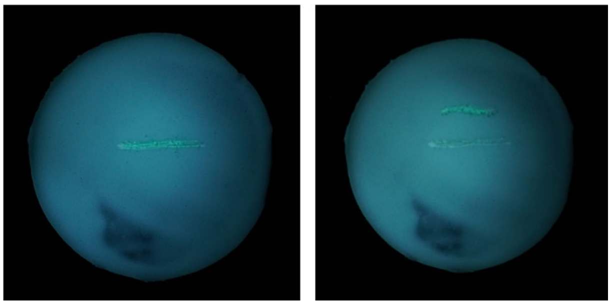

Figure S29. First writing and second writing of $(C_9NH_{20})_6[Pb_3Br_{12}]$ paper using a ceramic spatula, the images are captured under 365 UV light.
